# Zi Shen Wan Fang repaired blood–brain barrier integrity in diabetic cognitive impairment mice via preventing cerebrovascular cells senescence

**DOI:** 10.1186/s13020-024-01041-6

**Published:** 2024-12-19

**Authors:** Qingsheng Yin, Genhui Yang, Runtao Su, Jie Bu, Ying Li, Han Zhang, Yanjun Zhang, Pengwei Zhuang

**Affiliations:** 1 State Key Laboratory of Chinese Medicine Modernization, Tianjin University of Traditional Chinese Medicine, Jinghai District, Tianjin, 301617 China; 2 State Key Laboratory of Component-Based Chinese Medicine, Tianjin University of Traditional Chinese Medicine, Tianjin, 301617 China; 3 Haihe Laboratory of Modern Chinese Medicine, Tianjin University of Traditional Chinese Medicine, Tianjin, 301617 China; 4 Department of Integrated Rehabilitation, First Teaching Hospital of Tianjin University of Traditional Chinese Medicine, Tianjin, 300193 China; 5 National Clinical Research Center for Chinese Medicine Acupuncture and Moxibustion, Tianjin, 300193 China

**Keywords:** Diabetic cognitive impairment, Zi Shen Wan Fang, Blood–brain-barrier, Vascular senescence, Mangiferin

## Abstract

**Background:**

Blood–brain barrier (BBB) integrity disruption is a key pathological link of diabetes-induced cognitive impairment (DCI), but the detailed mechanism of how the diabetic environment induces BBB integrity disruption is not fully understood. Our previous study found that Zi Shen Wan Fang (ZSWF), an optimized prescription consisting of Anemarrhenae Rhizoma (*Anemarrhena asphodeloides Bge.*), Phellodendri Chinensis Cortex (*Phellodendron chinense Schneid.*) and Cistanches Herba (*Cistanche deserticola Y.C.Ma*) has excellent efficacy in alleviating DCI, however, whether its mechanism is related to repairing BBB integrity remains unclear. This study aims to reveal the mechanism of BBB integrity destruction in DCI mice, and to elucidate the mechanism by which ZSWF repairs BBB integrity and improves cognitive function in DCI mice.

**Methods:**

Diabetic mouse model was established by feeding a 60% high-fat diet combined with a single intraperitoneal injection of 120 mg/kg streptozotocin (STZ). DCI mice were screened with morris water maze (MWM) after 8 weeks of sustained hyperglycemic stimulation. ZSWF was administered daily at doses of 9.36 and 18.72 g/kg for 8 weeks. Cognitive function was evaluated using MWM, blood–brain-barrier (BBB) integrity was tested using immunostaining and western blot, the underlying mechanisms were explored using single-cell RNA sequencing (scRNA-seq), validation experiments were performed with immunofluorescence analysis, and the potential active ingredients of ZSWF against cerebrovascular senescence were predicted using molecular docking. Moreover, cerebral microvascular endothelial cells were cultured, and the effects of mangiferin on the expression of p21 and Vcam1 were investigated by immunofluorescence staining and RT-qPCR.

**Results:**

ZSWF treatment significantly ameliorated cognitive function and repaired BBB integrity in DCI mice. Using scRNA-seq, we identified 14 brain cell types. In BBB constituent cells (endothelial cells and pericytes), we found that *Cdkn1a* and senescence-associated secretory phenotype (SASP) genes were significantly overexpressed in DCI mice, while ZSWF intervention significantly inhibited the expression of *Cdkn1a* and SASP genes in cerebrovascular cells of DCI mice. Moreover, we also found that the communication between brain endothelial cells and pericytes was decreased in DCI mice, while ZSWF significantly increased the communication between them, especially the expression of PDGFRβ in pericytes. Molecular docking results showed that mangiferin, the blood component of ZSWF, had a stronger affinity with the upstream proteins of p21. In vitro experiments showed that high glucose significantly increased the expression of p21 and Vcam1 in bEnd.3 cells, while mangiferin significantly inhibited the expression of p21 and Vcam1 induced by high glucose.

**Conclusion:**

Our study reveals that ZSWF can ameliorate cognitive function in DCI mice by repairing BBB integrity, and the specific mechanism of which may be related to preventing cerebrovascular cells senescence, and mangiferin is its key active ingredient.

**Graphical Abstract:**

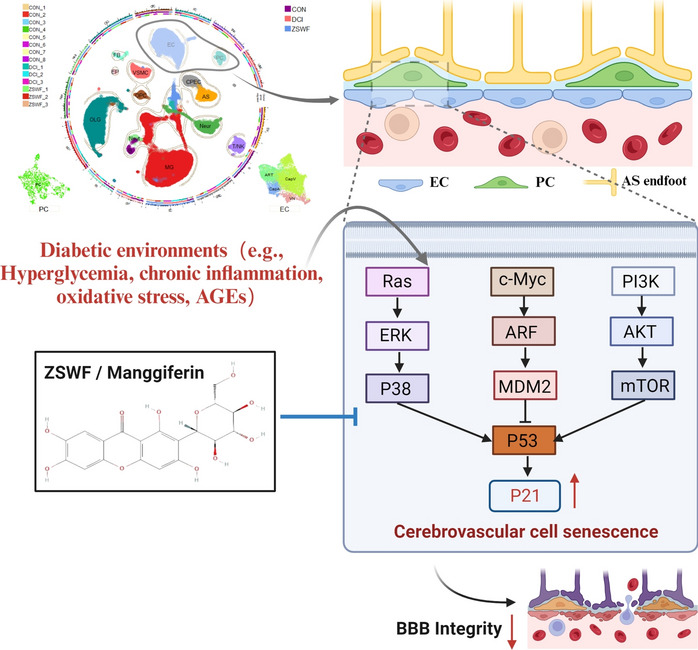

## Background

Cognitive dysfunction is an important central nervous system complication of diabetes. Previous epidemiological surveys have shown that 25–35% of diabetic patients are accompanied by cognitive dysfunction, and the probability of cognitive dysfunction in diabetic patients is 1.5–2.5 times higher than that in non-diabetic patients [1, 2]. Therefore, the development of drugs to prevent and treat DCI has been an arduous task for medical and health services. Unfortunately, despite enormous studies on DCI [3–5], its pathogenesis remains obscure and there are still no FDA approved drugs. Some multicenter clinical cohort studies using diabetes drugs to prevent cognitive impairment also showed that lowering or controlling blood glucose could not reduce the incidence of cognitive impairment [6–9], revealing the subsequent pathological mechanism mediated by hyperglycemia may be a potential target for the prevention and treatment of cognitive impairment.

BBB is a key interface separating circulating blood compounds from the brain and is essential for maintaining the brain microenvironment. Cumulative clinical and preclinical studies have shown that the diabetic environment mediates BBB integrity disruption and that BBB dysfunction is a key pathological link in DCI [10–13]. Some excellent studies have also revealed that repairing BBB integrity is a potential strategy to prevent and treat DCI [11, 14, 15]. However, given the ingenious physical composition and complex chemical structure of BBB, the mechanism by which diabetes leads to BBB integrity disruption remains unclear. The anatomical structure of the BBB is comprised of different fine cells, including endothelial cells (ECs), pericytes, and astrocytes, which presumably affect each other through reciprocal interactions [16]. scRNA-seq allows accurate analysis of the gene expression profles of different types of brain cells at the single-cell level, and has been widely used to analyze physiological and pathological signatures of BBB [17–22]. Therefore, scRNA-seq was used to explore the underlying mechanism of BBB integrity destruction in DCI mice. Senescence is a biological process defined by an apoptosis-resistant and irreversible arrested cell cycle with a distinct pro-inflammatory phenotype affecting neighboring cells [23]. Accumulated past studies have confirmed that senescence of cerebrovascular cells drives BBB integrity destruction, and targeted clearance of senescence vascular cells can prevent BBB dysfunction [24–26]. Most notably, past studies have also shown that cerebrovascular cell senescence is the pathological basis of diabetes-induced central nervous system complications [27–29]. Therefore, we will use the scRNA-seq technology to clarify the mechanism of BBB integrity destruction in DCI mice from the perspective of cerebrovascular cell senescence.

ZSWF is a prescription optimized under the guidance of DCI traditional Chinese medicine theory of “poison damage brain collaterals” pathogenesis and “clearing heat-fire and detoxifying” treatment, which consists of Anemarrhenae Rhizoma (*Anemarrhena asphodeloides Bge*), Phellodendri Chinensis Cortex (*Phellodendron chinense Schneid*) and Cistanches Herba (*Cistanche deserticola Y.C.Ma*). Our previous studies have repeatedly demonstrated that ZSWF can improve cognitive function in DCI mice, and investigated the potential mechanisms from the perspective of regulating kynurenine metabolism and intestinal flora [30, 31]. Moreover, we have previously investigated the distribution of ZSWF chemical components in the body, and found many prototype and metabolic components in the blood [32], suggesting that ZSWF may have direct vascular protective effects. Therefore, this study aims to clarify the potential mechanism by which ZSWF improves DCI from the perspective of protecting BBB integrity through scRNA-seq. Specifically, on the basis of confirming that ZSWF improves DCI, this study first investigated its protective effect on BBB integrity in DCI mice, and then analyzed the transcriptional characteristics of BBB constituent cells by using scRNA-seq. Furthermore, we applied previously identified ZSWF components into blood [32], screened the potential active components of ZSWF for anti-vascular senescence through molecular docking, and verified them by cultured cerebral microvascular endothelial cells in vitro. Overall, this study aims to uncover the underlying mechanism of BBB integrity in DCI mice and to provide new strategies for the development of innovative drugs against DCI.

## Methods

### Preparation of ZSWF extract

ZSWF is composed of Anemarrhenae Rhizoma (dried rhizome of *Anemarrhena asphodeloides* Bge.), Phellodendri Chinensis Cortex (dried bark of *Phellodendron chinense* Schneid.) and Cistanches Herba (dried fleshy stem of *Cistanche deserticola* Y.C.Ma) with a ratio of 1:1:1 in weight. Anemarrhenae Rhizoma and Phellodendri Chinensis Cortex were purchased from Anhui Bozhou Medical Materials Co., Ltd, and Cistanches Herba was purchased from Inner Mongolia Mandela Biotechnology Co., Ltd. These herbs were identified and processed by professors Tianxiang Li and Yan Wang from Tianjin University of Traditional Chinese Medicine, respectively. The detailed processing of these herbs is described previously [31]. At the end of processing, different concentrations of ZSWF extracts were prepared by reflux extraction, the detailed process was also described previously. In short, Anemarrhenae Rhizoma and Phellodendri Chinensis Cortex were mixed at a ratio of 1:1, and extracted with 8 times the amount of 80% ethanol for 3 times (2 h each time). The same weight of Cistanches Herba was extracted by reflux with 8 times of 80% ethanol for 3 times (2 h each time). After collecting the filtrate, the residue was extracted with 10 times of distilled water for 3 times (2 h each time). All filtrates were collected and concentrated into 0.936 and 1.872 g/ml solutions, respectively, and stored at −80 °C in the refrigerator.

### Animals and experimental design

120 C_57_BL/6J male mice (8–10 weeks) were purchased from Beijing Vital River Laboratory Animal Technology Co., Ltd (SCXK (Jing) 2016-0006) and housed under standard laboratory conditions (temperature 22 ± 2 °C, humidity 50 ± 15%, 12 h light/12 h dark cycle) in the SPF-level rearing room of the Laboratory Animal Center of Tianjin University of Chinese Medicine. All mice were allowed free access to water and feeding. All experimental procedures were approved by the Animal Ethics Committee of Tianjin University of Traditional Chinese Medicine (TCM-LAEC2020093, Tianjin, China).

To remove some of the animals unsuitable for the maze environment, all mice were subjected to the morris water maze (MWM, see the following for details) after 3 days of adaptive feeding. Then, the remaining qualified mice were randomly divided into control group and high-fat diet group. The control group (Con) was fed with ordinary diet, and the high-fat diet group was fed with 60% high-fat diet (containing 60% fat, 20% protein and 20% carbohydrate, Beijing Vital River Laboratory Animal Technology Co., Ltd.). After 3 weeks of high-fat diet, the type 2 diabetes model was induced by a single intrabitoneal injection of 120 mg/kg streptozotocin (STZ, S0130, Sigma, St Louis, MO, USA) as previously reported [31, 33]. Specifically, after the mice were fasted for 12 h, STZ was weighed away from light and dissolved using a pre-cooled 0.1 mmol/L sodium citrate buffer (pH = 4.5, Solarbio Technologies, Inc. Beijing, China). Subsequently, STZ solution with a concentration of 120 mg/kg was intraperitoneally injected in a light-free environment, and the injection volume was 0.1 mL per 10 g body weight of mice. Mice in the Con group were intraperitoneally injected with the same volume of sodium citrate buffer. After 1 week of STZ injection, all mice were tested for fasting blood glucose (FBG) by tail vein using a glucometer (Roche), and mice with FBG ≥ 11.1 mmol/L were screened as diabetic mice for subsequent experiments. Diabetic mice were then continued to be fed a high-fat diet for 8 weeks and screened using MWM, and mice with cognitive impairment were used for subsequent grouping and treatment. Finally, diabetic cognitive impairment mice were divided into three groups based on cognitive function: diabetic cognitive impairment group (DCI), ZSWF low dosage treatment group (ZSWFL, 9.36 g/kg, 2 times the clinical equivalent dosage) and ZSWF high dosage treatment group (ZSWFH, 18.72 g/kg, 4 times the clinical equivalent dosage). The dosage of ZSWFL and ZSWFH was selected based on the results of previous studies [30, 31]. Mice in the treatment group were orally gavaged with the corresponding dosage of ZSWF crude extract at 0.1 ml/10 g body weight once daily for 8 weeks, while mice in the DCI and Con groups were gavaged with an equal volume of distilled water. During the drug treatment, the treated and DCI groups continued to be fed with high-fat chow and the Con group was fed with normal chow. After the end of drug treatment, the body weight and fasting blood glucose of mice in each group were obtained, and MWM was used again to confirm the ameliorating effect of ZSWF on cognitive impairment in DCI mice. Then the experimental animals were deep anesthesia with isoflurane and executed, and hippocampus and whole brain were collected for the subsequent index tests.

### Morris water maze task

To further corroborate the effect of ZSWF on improving cognitive function in DCI mice, MWM experiments were performed according to previously reported procedure [34]. The Morris water maze consists of a round stainless steel pool with a diameter of 100 cm and evenly divided into 4 quadrants and a hidden platform with a diameter of 10 cm. The stainless steel pool is filled with water until the liquid level is 0.8 cm above the height of the hidden platform. Meanwhile, the stainless steel pool is evenly divided into 4 quadrants, namely east, west, south and north, and the letters E, W, S and N are marked in the center of the pool wall of the corresponding quadrant. Then one of the quadrants is defined as the target quadrant, where the hidden platform is placed in the center. During the whole process of the water maze experiment, the water temperature of the pool was kept at 23 ± 1 ℃, the visual cues around the maze were relatively fixed, the experimental environment was kept quiet, and the operator was fixed. To familiarize the experimental animals with the maze environment and reduce the stress response caused by stimuli such as water temperature, all mice were placed in a platformless maze environment to swim freely for 60 s the day before the water maze experiment began. During the positioning navigation experiments, the experimental mice were placed in water facing the pool wall from the opposite quadrant of the target quadrant, and the time the mice reached the hidden platform (escape latency) was recorded. If the mice did not find the hidden platform within 60 s, the escape latency was recorded for 60 s, and the mice were gently guided from the placed into the water quadrant to the platform, and allowed to remain on the platform for 15 s to remember the location of the platform and the environment around the platform. The positioning navigation experiment was performed 5 times, and the mice were gently wiped dry with dry gauze after each training. The escape latency and swimming trajectory of each mouse were recorded using the image automatic monitoring and processing system carried by MWM, and data from each group were analyzed using repeated measure ANOVA. To further evaluate the memory ability of the experimental mice, we also performed a space exploration experiment on the sixth day. In the space exploration experiment, a platform previously hidden underwater was removed and the mice were gently placed into a maze from the opposite quadrant of the target quadrant facing the pool wall and allowed to swim freely for 60 s. Then the time when the mice first arrived at the virtual platform, the frequency of crossing the virtual platform, the duration in the virtual platform quadrant, and the swimming trajectory were recorded using an automated image processing and analysis system, and the data were analyzed using one-way ANOVA.

### Immunohistochemistry

Immunofluorescence staining was performed to investigate the effect of ZSWF on BBB integrity and to verify the findings of scRNA-seq. The mice were sacrificed for cervical dislocation after deep anesthesia, and the whole brain tissue was separated and fixed with 4% paraformaldehyde. Gradient sucrose (10, 20, 30%) was then dehydrated and frozen sectioning was performed. A series of 10 μm coronal sections were cut from the anterior commissure to hippocampus with 100 μm interval for immunostaining. The functional and morphological alterations of BBB were assessed by IgG immunostaining [35]. Detailed procedures are as follows: After the frozen sections were placed at room temperature, the antigen-active clusters crosslinked by paraformaldehyde were repaired by a water bath at 95 ℃. 0.5% Triton-100 was used for membrane permeability and bovine serum albumin (BSA) was used to block non-specific antigenic sites. Cy3 conjugated Goat Anti-mouse IgG (H + L) was added for incubation, and the images were collected by fluorescence microscopy. Endothelial cell senescence and endothelial-pericyte communication were evaluated by double labeling of CD31 (Cat. No. ab281583, abcam; Cat. No. GB12063, Servicebio Technology) with P21 (Cat. No. GTX34925, GeneTex) and PDGFRβ (Cat. No. 3169S, CST), respectively. After the primary antibody was added and incubated at 4 ℃ overnight, the corresponding fluorescent secondary antibodies (goat anti-rabbit IgG H&L (Alexa Fluor® 488, Cat. No. ab150077, abcam) and Goat Anti-mouse IgG H&L (Alexa Fluor® 647, Cat. No. ab150115, abcam) were added and incubated at room temperature for 2 h, and the images were collected by fluorescence microscopy. Five random fields in the brain were photographed in each section, and the mander’s coefficients of colocalization was analyzed by Image J software.

### Western blot analysis

To investigate the effect of ZSWF on BBB integrity in DCI mice, the mice were sacrificed for cervical dislocation after deep anesthesia. The skull was then cut along the midline of the skull to expose the brain tissue, and hippocampus samples were collected and stored at −80 °C for testing. The total protein in hippocampus was extracted and quantified by BCA protein quantification kit according to the manufacturer’s protocal. Briefly, 20 mg of hippocampus sample was taken and cleaved with 100 μl lysate containing 10 μL PMSF, followed by centrifugation at 4 ℃ at 12,000*g* for 5 min, supernatant was collected, and protein concentration was determined using a BCA protein quantification kit (Cat. No.DB0028, Bioworld Technology). The total protein was then isolated by polyacrylamide gel electrophoresis (SDS-PAGE) and transferred to polyvinylidene difluoride (PVDF) membranes. The immunoblotted was then incubated overnight at 4 °C with primary antibody: anti-claudin 5 antibody (Cat. No. PA5-99415, Invitrogen Life Technology), anti-occludin antibody (Cat. No. 40-4700, Invitrogen Life Technology), anti-matrix metalloproteinase 9 antibody (MMP9, Cat. No. PA5-13199, Invitrogen Life Technology), and anti-β‑actin antibody (Cat. No. PA5-21396, Cell Signaling Technology). After 3 washes, the immunoblotted were incubated with the peroxidase-conjugated secondary antibody at room temperature for 2 h. Proteins were detected using the enhanced chemiluminescence detection kit (Millipore). Band intensities were quantified using Image J. Relative expression was normalized to β‑actin.

### Brain tissue dissociation for scRNA-seq

Brain tissue harvest and dissociation were performed at the same time (10:00–11:00) for each animal, thus limiting circadian variation [36]. The mice were sacrificed by cervical dislocation, brains were extracted, and hindbrain and olfactory bulb regions were removed. The remaining tissue was chopped on ice to less than 1mm cubic pieces, then enzymatically digested with 0.25% trypsin for 40 min, manually shaken every 5 min. Samples were then centrifuged at room temperature at 300 rcf for 30 s to remove the supernatant. Next, 1 × PBS (calcium and magnesium free) containing 0.04% weight/volume BSA (400 µg/ml) was added and then centrifugation at 300 rcf for 5 min. The cell pellet was resuspended in 1 mL red blood cell lysis buffer and incubated at 4 ℃ for 10 min to lysate red cells. Samples were then resuspended in 1 mL PBS containing 0.04% BSA. Next, samples were filtered over Scienceware Flowmi 40 µm cell strainers (VWR). After the brain cells were isolated, cell concentration and cell viability were measured by hematocytometer and Trypan blue staining. All cell lysates were stored on ice for no more than 1 h until sequencing.

### scRNA library preparation

Cell suspensions were loaded on a Chromium Single Cell Controller instrument (10× Genomics, Pleasanton, CA, USA) to generate single-cell gel beads in emulsions (GEMs). Then, scRNA-seq libraries were prepared with the Chromium Single Cell 3′ Library & Gel Bead kit v2 and i7 Multiplex kit (10× Genomics) according to the manufacturer’s instructions. Briefly, suspensions containing about 10,000 cells per sample were mixed with RT-PCR reaction before being added to a chromium chip already loaded with barcoded beads and partitioning oil. After the generation of GEMs, reverse transcription reactions were engaged to generate barcoded full-length cDNA, which was followed by disruption of emulsions using the recovery agent, and then cDNA clean-up was performed with DynaBeads Myone Silane Beads (Thermo Fisher Scientific). Next, cDNA was amplified by PCR for the appropriate number of cycles, which depended on the number of recovered cells. Subsequently, the amplified cDNA was fragmented, end-repaired, A-tailed, and ligated to an index adaptor, and then the library was amplified. Every library was sequenced on a Novaseq 6000 (Illumina), and 150 bp paired-end reads were generated.

### Single-cell sequencing data analysis

The Cell Ranger software pipeline (version 6.1.2) provided by 10× Genomics was used to map the raw reads of each sample to the mouse genome reference (version mm10) with default parameters. The filtered feature_bc_matrix produced by the Cell Ranger pipeline was used as a raw count matrix for each sample and processed separately in R (version, 4.3.0) for quality control (QC). In the QC process, the raw count matrix was read into the R package Seurat (v 4.3.0) [37] and was cleaned up with some criteria. Genes detected in less than 3 cells were excluded. Cells with less than 100 detected genes, or with less than 500 detected UMIs, or with more than 10% mitochondrial transcripts were also excluded. Then, doublets were detected and removed from the count matrix using the R package DoubletFinder (v2.0) [38], and ambient RNA was also detected and removed from the count matrix using the R package DecontX (v0.99) [39] to get a clean count matrix. After QC analysis and filtering, the clean count matrixes of all samples were merged and analyzed using the Seurat.

The merged count matrix was normalized using the NormalizeData function in Seurat. The top high variable genes were identified using the FindVariableGenes function in Seurat. The merged count matrix was then scaled using the ScaleData function in Seurat, with UMIs and the percentage of mitochondrial reads regressed. And then, the PCA analysis was performed using the RunPCA function in Seurat. The above 4 functions were carried out with default parameters. The R package Harmony (v0.1.1) was used to remove batches among the samples. After batch correction, the FindNeighbors function in Seurat was used to construct a shared nearest-neighbor graph among all cells, and the FindClusters function in Seurat was used to separate all cells into clusters. Different resolutions were tested when using FindClusters and the clustering results were evaluated with ROGUE [40] to help choose the most optimized resolution parameter for clustering cells. Clustered cells were transferred into a reduced dimensional space for easy visualization using both RunTSNE and RunUMAP functions in Seurat.

### Cell types and subtypes identification

The FindAllMarkers function in Seurat was used to identify marker genes of all clusters. Through comparing marker genes of each cluster to known cell type-specific marker genes that have been previously described in the literature or collected in the CellMarker database (http://bio-bigdata.hrbmu.edu.cn/CellMarker/), cell types were annotated manually for each cluster. And then, the cells of each cell type were re-clustered and separated into cell sub-types and annotated manually.

### Cell–cell communication analysis

The ligand or receptor was defined as “expressed” in a particular cell type if 10% of the cells of that type had non-zero read counts for the ligand/receptor encoding gene. Confident Ligand-Receptor interaction was then assessed by comparing their real average expression to a null distribution of average expression, which was generated by randomly selecting a pair of cell types and calculating the average expression of the Ligand-Receptor pair. Both CellPhoneDB (v2.0) [41] and CellChat (v1.6.1) were used to identify ligand-receptor interactions among cell types. The number of Ligand-Receptor pairs was used to define interaction strength between a pair of cell types. R packages igraph and Circlize were used to display the cell–cell communication networks.

### Molecular docking

Molecular docking was was employed to predict the potential active components of ZSWF anti-vascular cell senescence. Previously published ZSWF blood entry components [32] were defined as small molecules, while proteins upstream of the senescence marker p21 pathway (such as ERK, P38, mTOR, MDM2) were defined as target proteins. Molecular docking uses Discovery Studio 2019 software. The files of sdf. structure of ZSWF into blood compounds were downloaded from PubChem database, imported into Discovery Studio 2019 software to add CHARMm force field and hydrogen atoms, saved in pdb format. The three-dimensional crystal structure of the target protein was downloaded from the PDB protein database and Uniprot database. The water molecules in the crystal structure were removed, and amino acid residues around the original ligand were used as docking sites. Molecular docking binding energy reflects the binding strength of the active ingredient and protein, and the higher the binding energy, the better the binding ability.

### Cell culture and treatment

Mouse brain microvascular endothelial cells (bEnd.3) were purchased from Wuhan Xevill Biotechnology Co., LTD. The cells were cultured in low glucose DMEM (10% fetal bovine serum (FBS) and 1% penicillin/streptomycin) and incubated in an atmosphere of 5% CO_2_ and 95% humidified air at 37 °C. Cell viability was measured by CCK8 assay. bEnd.3 cells were inoculated into 96-well plates at a density of 1 × 10^4^ cells/ml, and treated with different concentrations of mangiferin (0, 25, 50, 100, 200, 400 µM) for 24 h, and then a final 10 μl of 10% CCK8 solution was added to each well with the plate protected from light. After 0.5 h, the optical density value was recorded at 450 nm using a microplate reader. Moreover, three groups of cells were created, and each group received the following treatment: cells did not receive any drug during the experiment (Con), bEnd.3 were incubated with 30 mM glucose for 48 h (High glucose), bEnd.3 were incubated with 30 mM glucose and 50 µM mangiferin simultaneously for 48 h (Mangiferin). After high glucose and drug incubation, cells were collected to fix and RNA was extracted for immunofluorescence staining and RT-qPCR, respectively.

### Immunofluorescence for bEnd.3

BEnd.3 cells were seeded in 24-well chamber slides at 50% confluency, fixed in 4% paraformaldehyde (biosharp). After washing with PBS, the slides were permeated with Triton X-100 (Beyotime) for 10 min, and blocked using 5% bovine serum albumin (BSA) and incubated overnight with p21 primary antibody (Cat. No. GTX34925, GeneTex) and vcam1 (Cat. No. ab171123, Abcam, Cambridge, UK). The cells were then incubated at 37 °C for 1 h with a goat-mouse IgG HL (Alexa Fluor 488) (Cat. No. Ab150113, Abcam, Cambridge, UK). The nuclei were stained with 4, 6-diamino-2-phenylindole (DAPI). Images were obtained using a fluorescence inverted microscope.

### Reverse transcription-quantitative (RT-q)PCR for bEnd.3

RT-qPCR was applied to assess the effects of mangiferin on markers of endothelial cell senescence induced by high glucose. Total RNA was isolated from bEnd.3 cells using Total RNA extraction kit (TIANGEN, China) in accordance with the manufacturer’s protocol. cDNA was synthesized using Prime Script™ RT kit (TIANGEN, China). Real-time quantitative PCR was established using TB Green Advantage qPCR premix (TIANGEN, SYBR Green, China) and real-time system PCR was performed (Bio RAD CFX96, America). Intron-spanning primers were designed based on the National Center for Biotechnology Information (NCBI) database and purchased from Shanghai Shenggong Biological Engineering Co., LTD. The relative expression of target genes was assessed using the 2^−ΔΔCq^ method and normalized to *Gapdh*. The following primers were used:

- *Cdkn1a* Forward 5′-AGCGCACAGGTAAGAGTGTTCATC-3′,
- Reverse 5′-AGCGCACAGGTAAGAGTGTTCATC-3′;
- *Gapdh*: Forward 5′-CATGAGAAGTATGACAACAGCCT-3′,
- Reverse 5′-AGTCCTTCCACGATACCAAAGT-3′.

### Statistical analysis

GraphPad Prism Software (version 5.0.2) and SPSS (version 17.0) were employed for data analysis and presentation. The MWM experimental data were represented by means ± SD, and the other results were presented as means ± SEM. The escape latency in the MWM experiment was analyzed using repeated measures ANOVA. We used a non-parametric two-sided Wilcoxon rank sum test in Seurat to identify DEGs and *P*_val_adjust was obtained by Seurat’s BH in all the comparisons discussed. The minimum threshold for significance was defined as *P*_val_adjust <0.05.

## Results

### ZSWF protected cognitive function in DCI mice

**Fig. 1:**
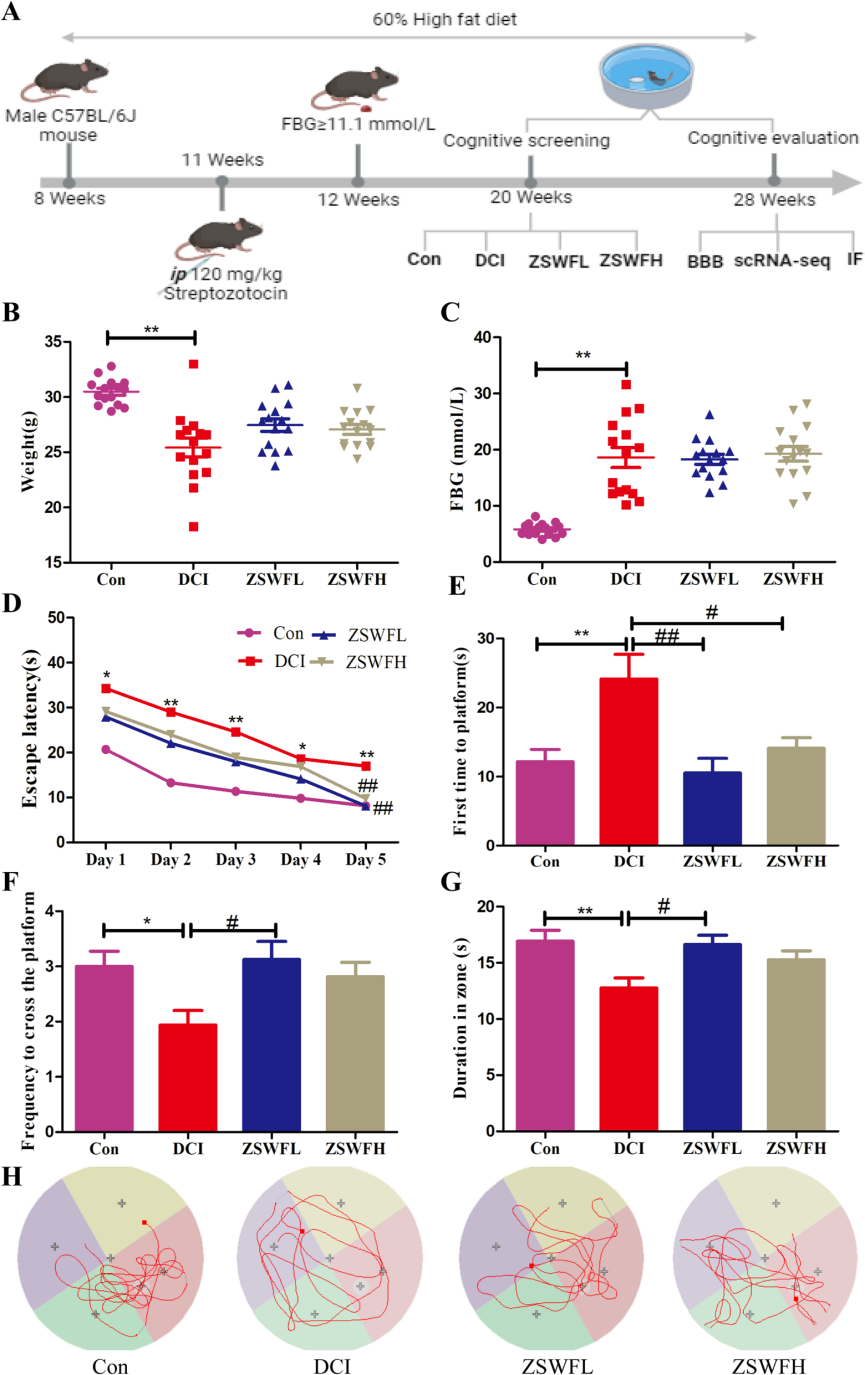
ZSWF protected cognitive function in DCI mice. **A** Overview of the experimental workflow. **B** body weight, **C** fasting blood glucose, **D** Escape latency, **E** time of first arrival at platform, **F** frequency of crossing the platform, **G** duration of the target quadrant, these data were expressed as the mean ± SD, (n = 15 for each group). Escape latency data were analyzed by repeated measurement ANOVA, other data were analyzed by one-way ANOVA, ^**^P < 0.01, ^*^P < 0.05 vs. Con group, ^##^*P* < 0.01, ^#^*P* < 0.05 vs. DCI group. **H** Representative swimming trajectory of each group of mice within 60 s in the space exploration experiment

To evaluate the protective effect of ZSWF on the cognitive function of DCI mice, a DCI mouse model was established, and the learning and memory ability of mice after ZSWF treatment was investigated using MWM, as shown in Fig. 1A. We first investigated the effects of ZSWF on body weight and FBG in DCI mice. At the end of treatment, our results showed that DCI mice had significantly reduced body weight (Fig. 1B) and significantly increased FBG (Fig. 1C), which is consistent with the clinical phenotype of type 2 diabetes patients. After treatment, both ZSWFL and ZSWFH tended to increase the body weight of DCI mice, but there was no statistical difference (*P* > 0.05) (Fig. 1B). For FBG, neither ZSWFL nor ZSWFH reduced FBG in DCI mice (*P* > 0.05) (Fig. 1C). The results of the positioning navigation test showed that the escape latency of mice in the DCI group was significantly increased compared to the Con group during the 5 consecutive days of training (Fig. 1D), while both ZSWFL and ZSWFH treated mice showed a significant reduction in escape latency by the 5th day of training (Fig. 1D), confirming that ZSWF can improve the learning ability of DCI mice. The results of the space exploration test showed that after platform removal, mice in the DCI group took significantly more time to reach the platform for the first time compared to the Con group (Fig. 1E) (*P* < 0.01), while the frequency of crossing the platform (Fig. 1F) and the time spent in the target quadrant (Fig. 1G) were significantly reduced (*P* < 0.05). Similarly, both ZSWFL and ZSWFH groups mice took significantly shorter time to first reach the virtual platform than mice in the DCI group (Fig. 1E) (*P* < 0.05). For the frequency of crossing the virtual platform and duration in the platform quadrant, our results showed that both the ZSWFL group and the ZSWFH group were higher than the DCI mice, but the ZSWFL group showed a statistical difference (Fig. 1F, G) (*P* < 0.05). These results confirmed that both ZSWFL and ZSWFH improved the learning ability of DCI mice, while ZSWFL seemed to have a better effect on memory ability. Moreover, we also observed the swimming trajectory of each group of mice within a specified time after platform removal. The results showed that the trajectories of DCI mice were directionless and aimless compared with the directional and regular trajectories of Con mice, while the trajectories of ZSWFL group and ZSWFH group mice also showed some directionality, as reflected by the fact that their swimming trajectories were mostly concentrated in the quadrant where the original platform was located (Fig. 1H). These results reaffirm potential of ZSWF to ameliorate cognitive function in DCI mice. Considering the fact that ZSWF has a weak effect on FBG in DCI mice, we speculate that the mechanism by which ZSWF ameliorates cognitive function in DCI mice may be the subsequent pathological response induced by hyperglycemia.

### ZSWF repaired BBB integrity in DCI mice

**Fig. 2:**
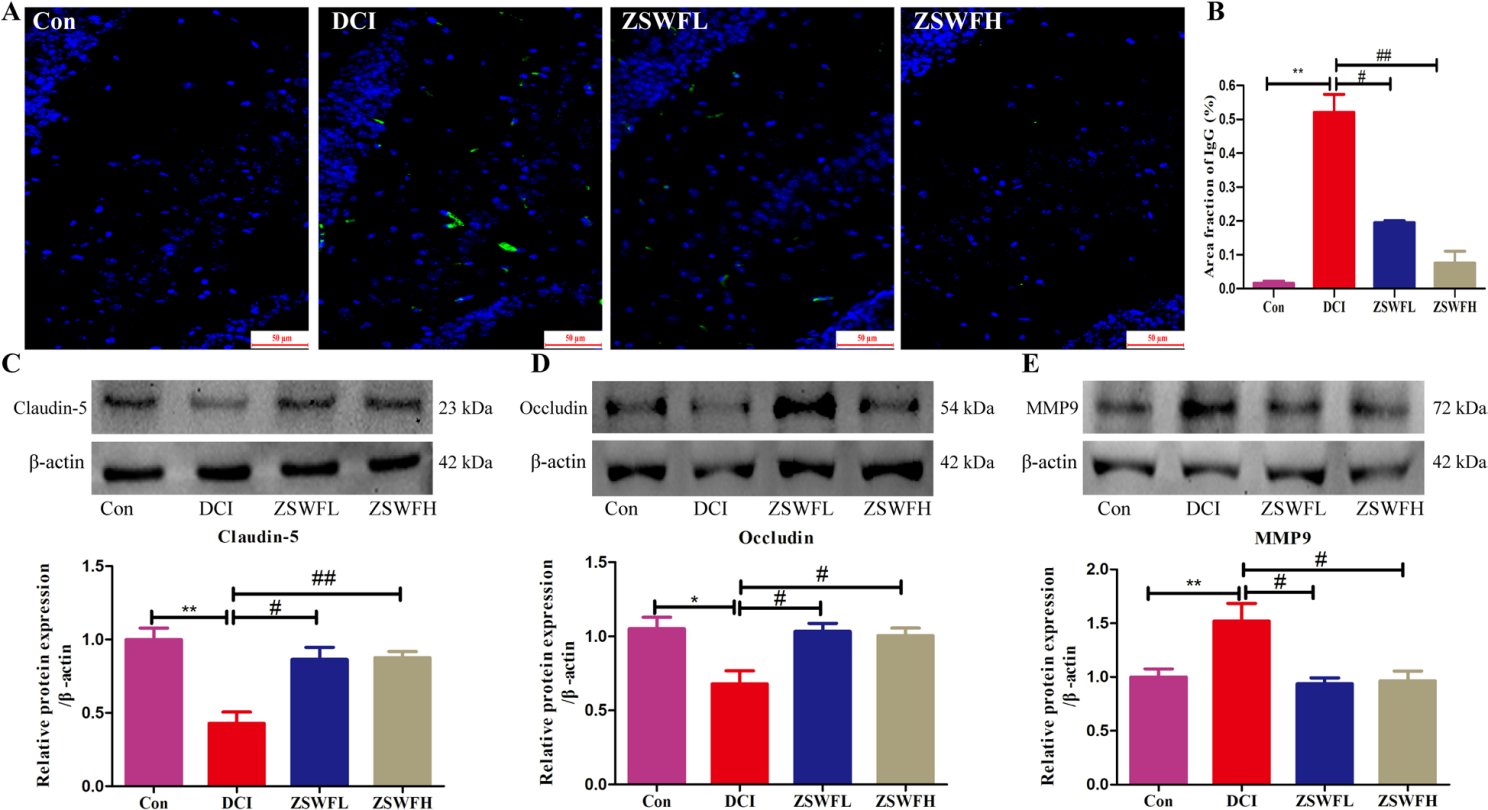
ZSWF repaired BBB integrity in DCI mice. **A** Representative IgG immunoactivity in hippocampus, **B** Statistics of the area fraction with IgG positive staining in hippocampal, these data were expressed as the mean ± SD, (n = 4 for each group). **C** Representative bands and expression levels of Claudin-5, **D** representative bands and expression levels of Occludin, **E** representative bands and expression levels of MMP9, these data were expressed as the mean ± SD, (n = 3 for each group). All data were analyzed using one-way ANOVA, ^**^P < 0.01, ^*^P < 0.05 vs. Con group, ^##^*P* < 0.01, ^#^*P* < 0.05 vs. DCI group

Considering the impact of the diabetic environment on BBB integrity and the critical role of BBB integrity on cognitive function, we thus investigated the repairing effect of ZSWF on BBB integrity in DCI mice. IgG leakage is a key indicator to evaluate the integrity of BBB. We first examined the repairing effect of ZSWF on BBB integrity in DCI mice using immunofluorescence staining, and the results showed an increase of IgG fluorescence intensity in the brain tissue of DCI group mice compared to Con group mice (Fig. 2A, B) (*P* < 0.01), while neither ZSWFL nor ZSWFH treatment reduced the IgG fluorescence intensity in the brain tissue of DCI mice (Fig. 2A, B) (*P* < 0.01), indicating that ZSWF repaired the BBB integrity of DCI mice. Moreover, we also performed western blot to detect the effect of ZSWF on the expression of hippocampal tight junction protein and matrix metalloproteinase 9 (MMP9) in DCI mice, and the results showed a significant decrease in Claudin-5 (Fig. 2C) (*P* < 0.01) and Occludin (Fig. 2D) (*P* < 0.05) expression and a significant increase in MMP9 (Fig. 2E) (*P* < 0.01) expression in DCI mice compared to the Con group. On the contrary, both ZSWFL and ZSWFH treatment significantly increased Claudin-5 and Occludin expression and decreased MMP9 expression in DCI mice (*P* < 0.05). These results suggest that ZSWF can repair damage to BBB integrity in DCI mice.

### Single-cell transcriptional landscape of DCI mouse brain treated with ZSWF

**Fig. 3:**
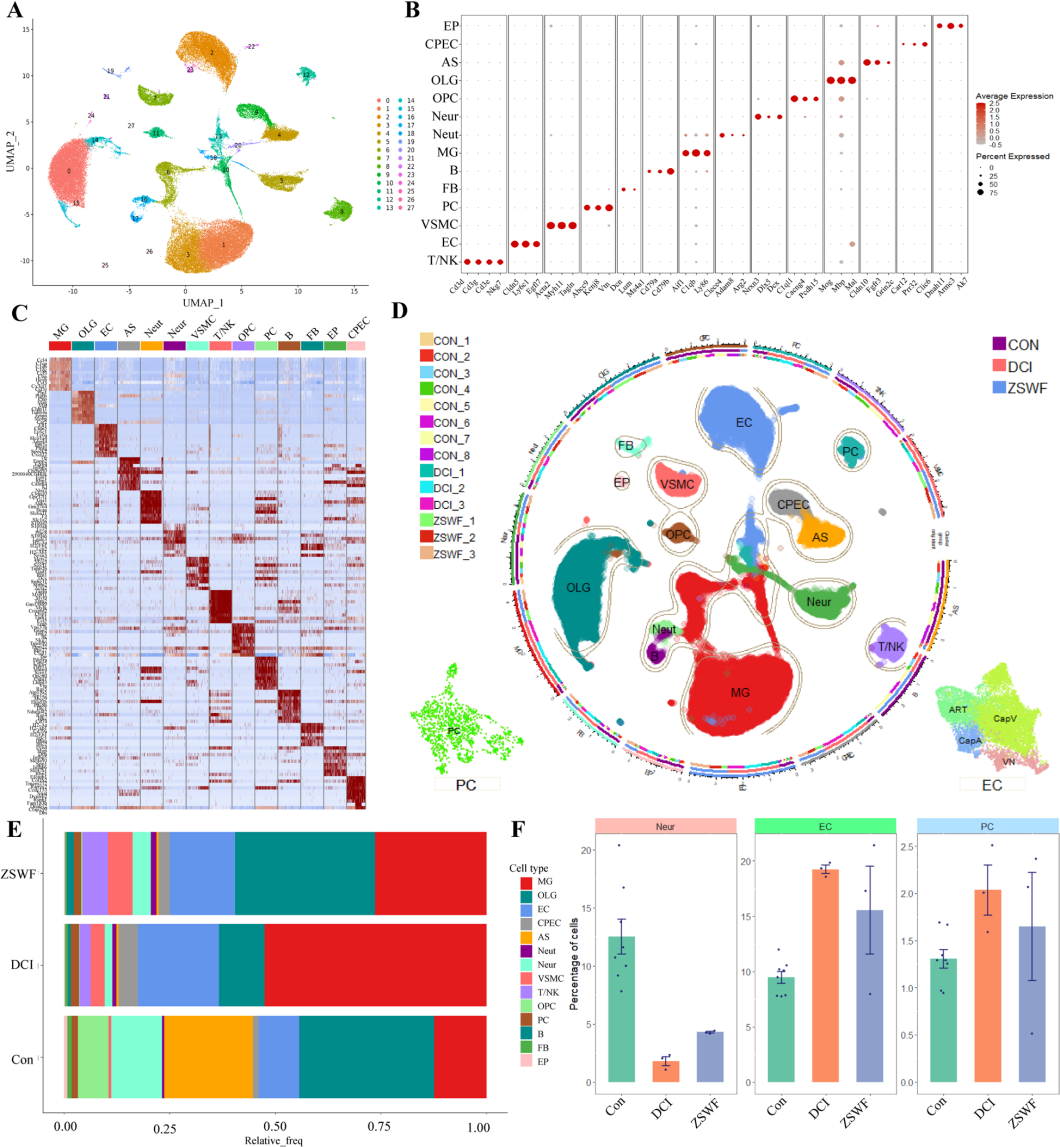
Single cell transcriptional landscape of DCI mouse brain treated with ZSWF. **A** Umap showing the clusters of all high-quality brain cells after reduction and clustering. **B** Dotplot showing the expression levels of well-known representative cell-type-enriched marker genes across all 14 cell types. **C** Heatmap showing the top ten most enriched genes for each cell type. **D** A total of 26,760 high-quality cells from 14 mouse brains in 3 different groups are projected by UMAP plot. Colors indicate the major brain cell types, and cluster boundaries are outlined by contour curve. The illustrations in the bottom two corners insets show endothelial cell subsets and pericytes maps. The axis outside the circular plot depicts the log scale of the total cell number for each cell class. The three colored tracks (from outside to inside) indicate mouse ID, group ID, and marker gene expression. The left legend shows the mouse ID/colors for the mouse ID track. The right legend shows groups ID/colors used for the groups track. **E** Bar chart showing the percentage of 14 brain cell types in the three groups. **F** Bar plot showing the percentage of neurons, vascular endothelial cells and pericytes in the three groups, these data were expressed as the mean ± SD of 8 Con, 3 DCI, and 3 ZSWF brains

Considering the cell diversity and heterogeneity of BBB structure, to depict the transcriptional signatures of DCI mice BBB destruction and identify the mechanism of ZSWF therapy, we performed scRNA-seq analysis on the brain tissues from three representative mice in DCI and ZSWF groups using 10× Genomics, the single cell transcriptome data of Con mouse brain were obtained from published public data [42]. After integration, batch removal and quality control, 26,760 cells high-quality cells were detected, among which 15,109, 26,471, and 23,180 cells were collected from the Con, DCI, and ZSWF groups, respectively. After unsupervised cell clustering and UMAP dimensionality reduction, 27 clusters with differentially expressed lineages were found (Fig. 3A). Using known markers for brain cell types, we identified 14 cell types, including neurons (Neur, *Nrxn3*, *Dlx5*, *Dcx*), astrocytes (AS, *Cldn10*, *Fgfr3*, *Grin2c*), microglia (MG, *Aif1, Ly86, C1qb*), oligodendrocytes (OLG, *Mog*, *Mbp*, *Mal*), oligodendrocyte progenitor cells (OPC, *Opcml, Vcan, Cacng4*), endothelial cells (EC, *Cldn5, Pecam1, Cdh5*), pericytes (PC, *Kcnj8, Abcc9, Vtn*), vascular smooth muscle cells (VSMC, *Acta2, Myh11, Tagln*), choroid plexus epithelial cells (CPEC, *Car12, Clic6, Prr32*), ependymal cells (EP, *Dnah11, Ak7, Armc3*), T/Natural killer cells (T/NK, *Cd3e, Cd3g, Cd3d, Nkg7*), B cells (B, *Ms4a1, Cd79a, Cd79b*), neutrophils (Neut, *Retnlg, Clec4e, Trem1*), and perivascular fibroblast-like cells (FB, *Dcn, Lum*) (Fig. 3B–D). The results of cell proportion among cell types showed that the ratio of MG, AS, OPC, and Neur was higher (Fig. 3E), which is consistent with previous reports [42–44], indicating that we obtained a reliable single-cell transcriptome atlas. The cell proportion between the groups showed that compared with the Con group, the proportion of Neur in the DCI mice brain decreased, while ZSWF treatment increased the proportion of Neur, which was consistent with the results of ZSWF improving the cognitive function of DCI mice, indicating that ZSWF has the potential to prevent neuron damage in DCI mice. The results of the proportion of BBB constituent cells showed that compared with the Con group, the proportion of cerebral vascular endothelial cells and pericytes in DCI mice significantly increased (Fig. 3F), among which endothelial cells were consistent with the previously reported results of cerebrovascular neovasculation induced by diabetes environment [45, 46], while ZSWF reduced the proportion of endothelial cells and pericytes (Fig. 3F), preliminarily suggested that endothelial cells and pericytes may be the target cells of ZSWF to improve DCI.

### ZSWF prevented brain endothelial cells senescence in DCI mice

**Fig. 4:**
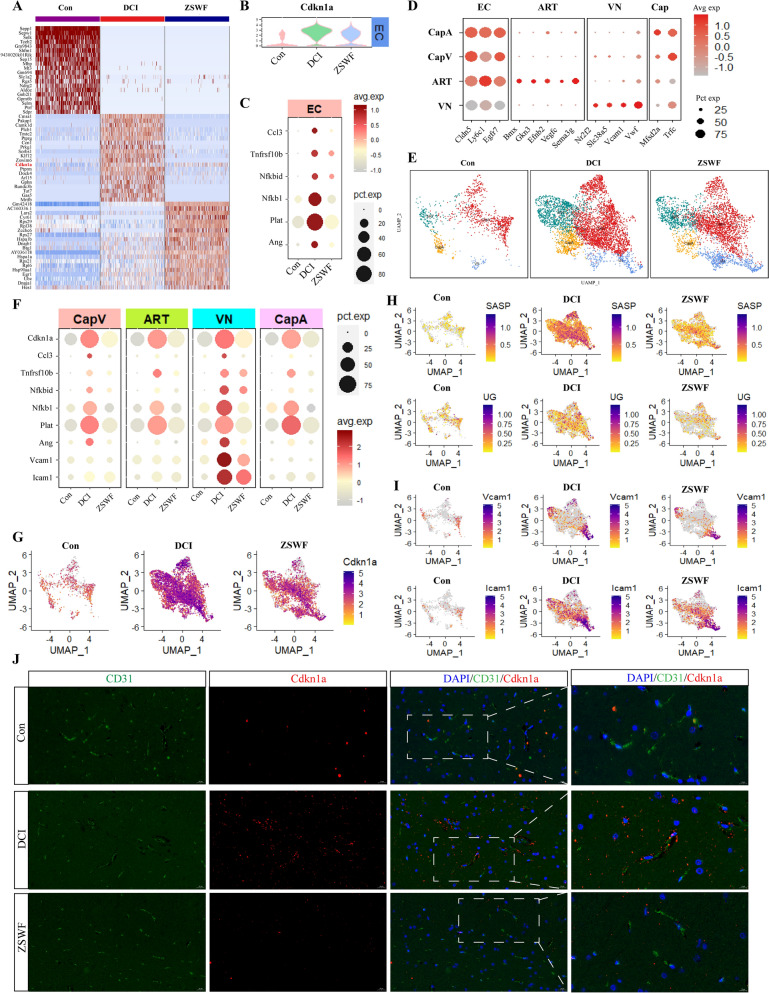
ZSWF prevented brain endothelial cells senescence in DCI mice. **A** Heatmap showing the top 20 most enriched genes for each gruop. **B** Violin plot showing the distribution of expression levels of *Cdkn1a* in endothelial cells of each group. **C** Dotplot showing the expression levels of SASP genes (such as *Ccl3, Tnfrsf10b, Nfkbid, Nfkb1, Plat,* and *Ang*) in endothelial cells of each group, the colors on the right of the picture represent the average expression level of these genes, and the circles represent the percentage distribution of these genes in each group of cells. **D** Dotplot showing the expression levels of well-known representative marker genes in vascular endothelial cells. **E** Umap showing the distribution of endothelial cell subtypes (ART, arterial endothelial cell; VN, venous endothelial cell; CapA, capillary arterial endothelial cell; CapV, capillary venous endothelial cell) in each group. **F** Dotplot showing the expression levels of SASP genes (such as *Ccl3*, *Tnfrsf10b*, *Nfkbid*, *Nfkb1*, *Plat*, *Ang*, *Vcam1*, and *Icam1*) in each endothelial cell subtype of three groups. **G** Umap showing the distribution of expression levels of *Cdkn1a* in endothelial cell subtype of each group. **H** Umap showing the expression levels of SASP genes set (such as *Ccl3, Tnfrsf10b, Nfkbid, Nfkb1, Plat,* and *Ang*) and senescence upstream genes set (such as *Kra, Map2k6, Mapk14,* and *Foxo1*) in endothelial cell subtype of each group. **I** Umap showing the expression levels of leukocyte adhesion molecules (*Vcam1* and *Icam1*) in endothelial cell subtype of each group. **J** Immunofluorescence depicting the expression of senescence signature proteins (Cdkn1a, red) in endothelial cells (CD31, green) of each group (scale bar, 20 µm)

To further elucidate the molecular mechanism of BBB integrity destruction in DCI mice and the intervention mechanism of ZSWF, we then investigated the transcriptional characteristics of endothelial cells in DCI mice and after ZSWF treatment. The transcription profiles of mouse endothelial cells in each group were shown in Fig. 4A, which showed significantly different gene expression characteristics. Notably, we found that the expression of cyclin dependent kinase inhibitor 1A (*Cdkn1a*, also known as *p21*), a senescence-related gene, was significantly elevated in endothelial cells of DCI mice, while ZSWF treatment significantly decreased the expression of *Cdkn1a* in endothelial cells (Fig. 4A, B). This results preliminarily suggested that endothelial cells of DCI mice showed senescence phenotype, and ZSWF could prevent the senescence of DCI endothelial cells. Inspired by this phenomenon, we then investigated the changes of senescence-associated secretory phenotype genes (such as *Ccl3, Ccl4, Ang, Tnfrsf10b, Nfkbid, Nfkb1, Plat*) in DCI mice brain endothelial cells, and found that SASP genes expression were significantly increased in DCI mice endothelial cells, suggesting that DCI brain endothelial cells showed a senescence phenotype (Fig. 4C). Fortunately, our results also showed that ZSWF treatment significantly improved the senescence phenotype of endothelial cells in DCI mice.

Furthermore, to further investigate whether there is structural heterogeneity in senescence of brain endothelial cells in DCI mice, using the known subsets of ECs makers, we divided them into four subsets, namely arterial ECs (ART, *Bmx, Gkn3, Efnb2, Vegfc*), venous ECs (VN, *Nr2f2, Slc38a5, Vwf*), capillary arterial ECs (CapA, *Mfsd2a, Trfc, Efnb2*) and capillary venous ECs (CapV, *Mfsd2a, Trfc, Vwf*) (Fig. 4D, E). Our results showed that *Cdkn1a* and SASP related genes were highly expressed in all types of ECs in DCI mice, while ZSWF treatment significantly reduced senescence and its secretory phenotype in all types of ECs in DCI mice (Fig. 4F–H). Notably, two senescence-associated secretory genes involved in leukocyte adhesion, such as *Vcam1* and *Icam1*, were specifically highly expressed in DCI mice venous ECs, and ZSWF treatment significantly increases their expression, suggesting that ZSWF also prevents leukocyte migration into the brain of DCI mice (Fig. 4F, I). Moreover, our results observed that ZSWF significantly reduced the expression of *Cdkn1a* upstream signaling molecules (for example, *Kras*, *Map2k6*, *Mapk14,* *Myc,* *Arf1,* *Pikc3,* *Akt1,* *mTOR,* and *Foxo1*) (Fig. 4H). Finally, to further verify the transcriptional results, we used immunofluorescence to detect the expression of Cdkn1a in ECs, and fortunately found that compared with the Con group, the expression of Cdkn1a in brain ECs and adjacent regions of DCI mice was increased, while ZSWF treatment significantly decreased the expression of Cdkn1a in DCI mice ECs (Fig. 4J). In conclusion, these results suggest that DCI mice brain ECs exhibit senescence phenotype, and ZSWF can prevent ECs senescence in DCI mice.

### ZSWF prevented pericyte senescence in DCI mice

**Fig. 5:**
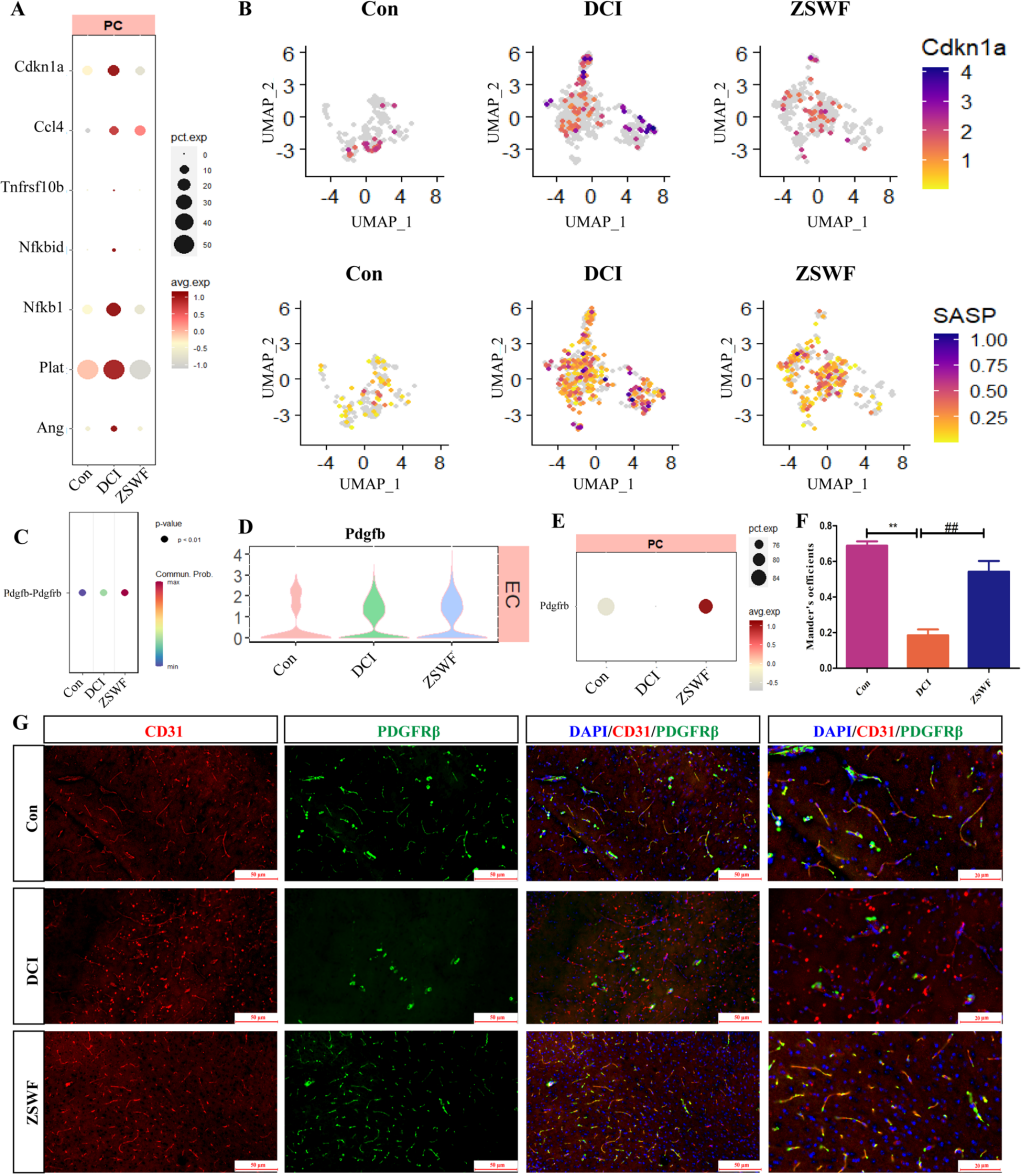
ZSWF prevented pericyte senescence in DCI mice. **A** Dotplot showing the expression levels of SASP genes (such as *Ccl3, Tnfrsf10b, Nfkbid, Nfkb1, Plat,* and *Ang*) in pericytes of each group. **B** Umap showing the expression levels of Cdna1a and SASP genes set (such as *Ccl3, Tnfrsf10b, Nfkbid, Nfkb1, Plat,* and *Ang*) in pericytes of each group. **C** Dotplot showing the communication intensity of Pdgfb-Pdgfrβ receptor and ligand pairs between endothelial cells and pericytes in each group. **D** Violin plot showing the distribution of expression levels of *Pdgfb* in endothelial cells of each group. **E** Dotplot showing the expression levels of *Pdgfrβ* in pericytes of each group. **F** Histogram showing the colocalization coefficients of endothelial cells and pericytes, these data were expressed as the mean ± SD, n = 4. **G** Immunofluorescence depicting the colocalization of endothelial cell (CD31, green) and pericytes (PDGFRβ, red) of each group (scale bar, 20 µm)

Pericytes connected on the parenchymal side of endothelial cells are also key components of BBB. Therefore, we also investigated the transcriptional signatures of PCs in DCI mice and the effects of ZSWF treatment on them. Similarly, our results showed that senescence-related signature and SASP genes were also significantly elevated in PCs of DCI mice (Fig. 5A, B), indicating that PCs of DCI mice also showed an senescence phenotype. Fortunately, *Cdkn1a* and senescence related secretory phenotype genes in PCs of mice treated with ZSWF were significantly reduced compared with DCI group (Fig. 5A, B), suggesting that ZSWF prevented PCs senescence in DCI mice. Furthermore, we investigated the key communication relationships between ECs and PCs in DCI mice. Notably, our results showed that ZSWF significantly increases the communication intensity of *Pdgfb-Pdgfrβ* signals (Fig. 5C). Vascular ECs derived platelet-derived growth factor B (*Pdgfb*) can bind to *Pdgfrβ* on the PCs surface, which is crucial for the recruitment of PCs to adhere to the vascular surface and maintain the integrity of BBB. Further expression analysis showed no significant difference of ECs-derived *Pdgfb* between the three groups (Fig. 5D), while *Pdgfrβ* expression in PCs was significantly reduced in DCI mice brains (Fig. 5E). Fortunately, ZSWF treatment significantly increased the expression of *Pdgfrβ* in DCI mice brain PCs (Fig. 5E), we thus speculate that ZSWF can restore BBB integrity in DCI mice by recruiting PCs coverage. To verify this hypothesis, we used immunofluorescence co-localization to analyze PCs coverage of ECs. Our results also confirmed that PDGFRβ co-localization with ECs was significantly reduced in the brains of DCI mice, while ZSWF treatment significantly increased the co-localization of ECs and PDGFRβ (Fig. 5D, E). These results suggest that ZSWF maintains BBB integrity in DCI mice by preventing PCs senescence and repairing ECs-PCs communication.

### Mangiferin prevents endothelial cell senescence induced by high glucose

**Fig. 6:**
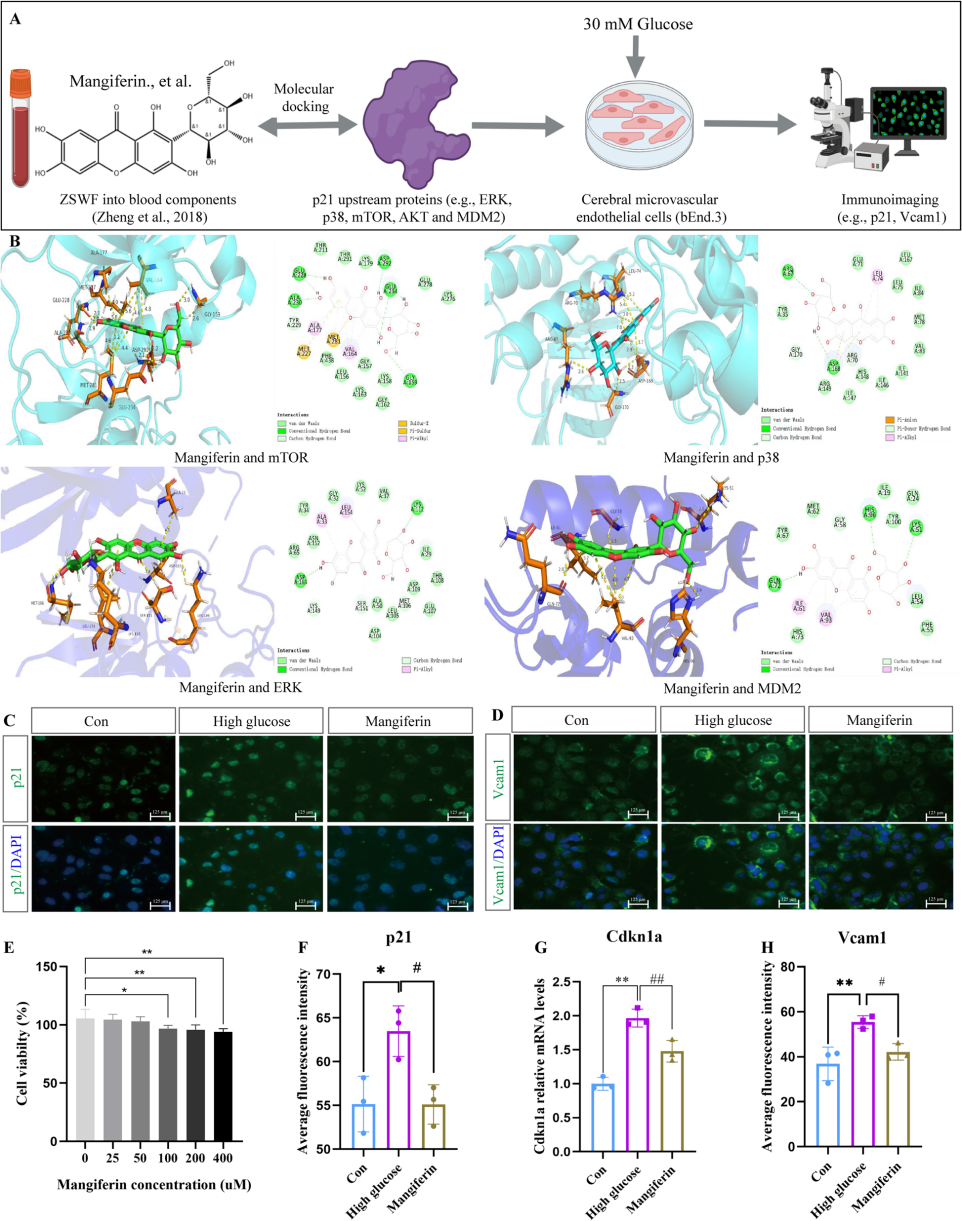
Mangiferin prevents endothelial cell senescence induced by high glucose. **A** Overview of ZSWF anti-vascular senescence active ingredient screening workflow. **B** Pattern diagram of molecular docking, including mangiferin and mTOR, p38, ERK and MDM2, respectively. **C** Representative fluorescence imaging of p21 protein expression. **D** Representative fluorescence imaging of Vcam1 protein expression. **E** Histogram represents the effects of mangiferin at different concentrations on the cytotoxicity of bEnd.3, this data was expressed as the mean ± SD and analyzed using t-test, n = 6, ^**^P < 0.01, ^*^P < 0.05 vs. 0 µM. **F** Histogram shows the protein expression level of p21. **G** Histogram shows the mRNA expression level of *Cdkn1a*. **H** Histogram shows the protein expression level of Vcam1. These data were expressed as the mean ± SD, these data were analyzed using one-way ANOVA, n = 3, ^**^P < 0.01, ^*^P < 0.05 vs. Con, ^##^*P* < 0.01, ^#^*P* < 0.05 vs. High glucose

On the basis of demonstrating that ZSWF can repair the integrity of the BBB in DCI mice by preventing cerebrovascular cell senescence, to further reveal the potential chemical composition of ZSWF against vascular senescence, we performed active ingredient screening using molecular docking and in vitro cell assay validation (Fig. 6A). Using the previously reported ZSWF blood chemical components [32] to perform molecular docking with p21 upstream signaling proteins (e.g. mTOR, p38, ERK, and MDM2), we found that mangiferin, a component derived from the Anemarrhenae rhizoma-Phellodendri chinensis cortex pair, has a stronger binding energy with the upstream proteins of p21 than other components (Fig. 6B, Table 1), suggesting that mangiferin may be a potential active component of ZSWF in anti-vascular senescence. Furthermore, to further confirm the discovery of molecular docking, we cultured bEnd.3 cells in vitro with medium containing normal glucose (5 mM) and high glucose (30 mM), and treated with mangiferin. Cytotoxicity tests showed that 50 µM mangiferin presented a safe dosage (Fig. 6E). Immunostaining results showed that, compared with Con group, high glucose stimulation significantly increased the expression of cell senescence marker p21 protein in bEnd.3 cells, while mangiferin treatment significantly inhibited the expression of p21 (Fig. 6C, F). RT-qPCR results also showed that mangiferin significantly decreased Cdkn1a mRNA expression in bEnd.3 cells induced by high glucose (Fig. 6G), indicating that high glucose stimulation can accelerate endothelial cell senescence, and mangiferin has anti-endothelial cell senescence effect. Meanwhile, we also examined the effect of high glucose stimulation on Vcam1 protein expression and mangiferin intervention in b.End3 cells. Our results also showed that high glucose stimulation significantly increased the expression of Vcam1 protein in endothelial cells, while mangiferin treatment significantly decreased the expression of Vcam1 protein induced by high glucose (Fig. 6D, H). These results suggest that mangiferin can indeed prevent cerebral vascular senescence induced by high glucose, and is one of the key active components of ZSWF to protect BBB integrity in DCI mice.

**Table 1:** Binding energy of ZSWF into blood components and P21 upstream proteins

CDOCKER energy (Kcal/mol)	mTOR	p38	ERK	MDM2
Mangiferin	25.0096	19.3859	28.6698	16.5056
Armepavine	21.7576	18.8355	24.5489	17.0896
Isomangiferin	18.2643	19.3885	22.5182	12.3331
Lotusine	17.545	14.3731	18.5929	9.43675
Phellodendrine	17.858	7.82887	17.4037	11.1723
Tembetarine	19.6809	7.91893	16.4526	8.89275
Magnoflorine	5.57458	0.6692	8.04119	−4.23999
Jatrorrhizine	4.73411	9.92541	5.4543	0.40794
Phellamurin	−3.34497	−23.4787	5.18335	−7.66213
Oxyberberine	0.23961	2.50728	2.75612	−1.42216
Menisperine	−1.08088	−9.00297	−0.7990	−7.96665
berberrubine	−5.43259	−3.81597	−8.05427	−10.2438
Berberastine	−10.8052	−7.26413	−8.73267	−14.5512
Thalifendine	−7.4879	−5.60026	−9.00103	−14.8097
Berberine	−12.2187	−9.53648	−12.852	−15.3749

## Discussion

Cognitive dysfunction is a common central nervous system complication of diabetes mellitus, the incidence of which increases year by year and seriously affects the quality of life of patients. Limited by the complex and not fully understood pathological mechanisms of DCI, there are still no FDA-approved treatments. More unfortunately, past multi-center clinical studies have found that neither metformin nor insulin replacement therapy can effectively reduce the incidence of DCI [8, 9], revealing that single-target drugs to control or reduce blood glucose are not ideal for the prevention and treatment of DCI, and the development of multi-target combination drugs may be a new trend for the prevention and treatment of DCI in the future. Multi-component traditional Chinese medicine has unique advantages in the treatment of complex diseases. Our previous studies have investigated and found that ZSWF has a good effect on improving cognitive impairment in DCI mice, and preliminarily explained the mechanism from the perspective of regulating kynurenine metabolism and maintaining intestinal flora [30, 31]. Notably, our previous studies also found that ZSWF has many prototypic and metabolic components that enter the blood, suggesting that it may have a direct vascular protective effect. In this study, therefore, on the basis of confirming the efficacy of ZSWF to improve DCI, we focused on the BBB perspective and employed scRNA-seq technology to investigate the mechanism of ZSWF to improve DCI. Fortunately, our findings not only revealed a new pathological mechanism of DCI in which diabetes accelerates cerebrovascular senescence, induces BBB integrity destruction and worsens cognitive impairment, but also clarified a new pharmacological mechanism of ZSWF to repair BBB integrity in DCI mice through anti-cerebrovascular senescence, providing a new strategy for DCI prevention and treatment from the perspective of anti-cerebrovascular senescence.

To further confirm the efficacy outcome of ZSWF in improving DCI, the DCI mouse model was established according to the previous method and the therapeutic effect of ZSWF was investigated. Consistent with previous results, the current study reaffirms that ZSWF can significantly reverse cognitive function in DCI mice. Considering that hyperglycemia is the initial pathological factor of DCI, the study first investigated the hypoglycemic effect of ZSWF. Unfortunately, our results show that ZSWF does not reduce FBG in DCI, suggesting that a subsequent pathological cascade mediated by hyperglycemia may be the target of ZSWF.

BBB is an interface that separates the central nervous system from the peripheral circulation, composed of microvascular ECs, PCs, and astrocytes, and is a key barrier to avoid damage to the brain from potentially neurotoxic and inflammatory agents in the circulation, such as cytokines, erythrocytes, leukocytes and pathogens [47]. However, BBB is susceptible to the diabetic environment, such as hyperglycemia, hyperlipidemia, and chronic inflammation [12, 48]. Accumulated past studies have shown that BBB integrity disruption mediated by homeostasis imbalance of peripheral circulation in diabetes is an early hallmark of cognitive impairment [11]. Therefore, the study investigated the effect of ZSWF on BBB integrity in DCI mice. According to the previously reported method [49], we evaluated BBB integrity by IgG staining. The results showed that ZSWF treatment significantly reduced the IgG positive staining in hippocampus, suggesting that ZSWF can repair BBB functional integrity in DCI mice. Tight junction proteins (such as Occludin and Claudin-5) that adhere to endothelial cells are essential for maintaining the structural integrity of BBB. Therefore, we also investigated the effect of ZSWF on the expression of tight junction proteins in DCI mice, and fortunately found that ZSWF treatment significantly increased the expression of brain tight junction proteins in DCI mice. MMP9 is a zinc-dependent protease that degrades many structural components of the extracellular matrix and non-extracellular matrix proteins [50]. Studies have shown that the expression of MMP9 is closely related to BBB integrity, and the diabetic environment can mediate the destruction of BBB by promoting the expression and secretion of MMP9 in pericytes [51]. Therefore, we examined the expression of MMP9 protein and found that ZSWF treatment significantly reduced the expression of MMP9 protein in the hippocampus of DCI mice. These results suggest that ZSWF restored BBB integrity in DCI mice.

ScRNA-seq provides an alternative method to study the cellular heterogeneity of the brain [52–54]. Compared with the average changes of various types of cells obtained by bulk RNA sequencing, scRNA-seq can accurately capture the transcriptional characteristics of tens of thousands of individual cells, and has made great achievements in analyzing the pathological mechanisms of a variety of complex diseases [42, 43, 55, 56]. More importantly, scRNA-seq also reflects a wide range of application scenarios for the analysis of complex mechanisms of traditional chinese medicine [57–60]. For example, fan et al. used scRNA-seq to reveal the synergistic mechanism of Shenmai injection combined with PD-1 inhibitors to improve non-small cell lung cancer by enhancing antitumor immunity [60]. Therefore, to reveal the mechanism of BBB integrity destruction in DCI mice and investigate the repair mechanism of ZSWF, we performed scRNA-seq on mouse brain tissues in the DCI group and the ZSWF treatment group, and obtained single-cell data of control mice from a public database (GSE129788). By integrating and quality controlling these data using R packets and identifying brain tissue cell types using known markers in brain tissue, 14 common brain cell types were identified, including neurons, astrocytes, microglia, and vascular-associated cells. The intercellular ratio results showed that neurons, microglia, and astrocytes accounted for a high proportion of all brain cells, which is similar to the previously published results for brain cell types [42, 61, 62], indicating that we obtained reliable single-cell transcriptome data. The results of cell proportion between the groups showed a decreased proportion of neurons in DCI mice, which was also consistent with the previously reported results of DCI neuron apoptosis [31]. The results of the proportion of BBB constituent cells showed that the proportion of ECs and PCs in the brain of DCI mice was significantly increased compared with that of Con group, and this result was also supported by diabetes induced cerebrovascular neovasculation [45, 46]. Fortunately, these abnormalities were reversed by ZSWF, providing initial clues that ZSWF improves cognitive function by targeting cerebrovascular cells to prevent neuronal apoptosis. Furthermore, our results also found that the proportion of microglia in the brain of DCI mice was significantly increased, while ZSWF could significantly reduce the proportion of microglia, suggesting that microglia activation may also play a crucial role in the pathological progression of DCI, and may also be the target cell of ZSWF to improve DCI. Since our current study focused on investigating the transcriptional signatures of cerebrovascular cells, the transcriptional characteristics of microglia will be analyzed in depth in our subsequent studies.

Inspired by the above clues, we focused on investigating the transcriptional characteristics of vascular-associated cells. Fortunately, we found that the expression of *Cdkn1a*, a landmark of senescent cells, was significantly increased in the brain endothelial cells of DCI mice. Accumulated past studies have also shown that *Cdkn1a* is involved in the senescent of multitudinous cells [63, 64], including vascular ECs [65–67]. Our current study revealed that *Cdkn1a* is highly expressed in almost all types of endothelial cells and that ZSWF significantly reduces *Cdkn1a* expression, suggesting that ZSWF has the potential to prevent endothelial cell senescence in DCI mice. Senescent cells usually secrete numerous factors, including pro-inflammatory cytokines, chemokines, and angiogenic factors, which are known as SASP. SASP can not only mediate the pathophysiological effects of senescent cells themselves, but also promote the communication between senescent cells and surrounding cells, thus affecting the function of neighboring cells. In this study, we found that chemokine *Ccl3* and pro-inflammatory factors such as *Tnfrsf10b*, *Nfkbid*, and *Nfkb1* were significantly overexpressed in DCI endothelial cells, while ZSWF significantly reduced the expression of these factors. *Ccl3* is a key factor that promotes transendothelial migration of T cells [68], which suggests that there may be a large number of infiltrating T lymphocytes in DCI brains, and ZSWF has the potential to prevent T cell infiltration. The high expression of proinflammatory factors reveals that DCI brain endothelial cells have a hyperinflammatory response, and importantly, endothelial inflammation is also a key cause of BBB integrity destruction [69], which suggests that ZSWF can repair BBB integrity in DCI mice by preventing endothelial cell senescence and reducing endothelial cell inflammation. Benefiting from the development of scRNA-seq, brain ECs have also been divided into different subtypes, including arterial endothelial cells, venous endothelial cells, and capillary endothelial cells. To further investigate the heterogeneity of DCI senescent endothelial cells and SASP, we used known markers to classify endothelial cells into arterial endothelial cells, venous endothelial cells, capillary arteries endothelial cells, and capillary veins endothelial cells. Our results showed that *Cdkn1a* and SASP genes were highly expressed in almost all types of endothelial cells in DCI mice, while ZSWF significantly reduces the expression of these genes. The only notable thing is that we found that two molecules involved in leukocyte adhesion, *Icam1* and *Vcam1*, are only characteristically highly expressed in venous ECs, which is consistent with the previously reported results that these adhesion molecules are expressed exclusively in venous endothelial cells [18]. Meanwhile, this result also suggests that DCI brain may infiltrate a large number of leukocytes, and ZSWF can inhibit the infiltration of cerebral leukocytes in DCI mice.

On the basis of demonstrating the senescence of cerebral vascular ECs in DCI mice and the inhibitory effect of ZSWF, the study then investigated whether the PCs of DCI mice were senescence and the effect of ZSWF. Fortunately, our results also confirmed that DCI mice brain PCs also showed a senescence phenotype, and ZSWF inhibited PCs senescence. Furthermore, we investigated the communication between senescent ECs and PCs in the DCI brain and found that ZSWF significantly increased the *Pdgfb-Pdgfrβ* signal strength. ECs-derived *Pdgfb* is essential for the recruitment of PCs by interacting with *Pdgfrβ*, and the decreased expression of perivascular *Pdgfrβ* reflects the loss of PCs, which is fatal to the maintenance of BBB integrity [70, 71]. Overall, our scRNA-seq data not only revealed that cerebrovascular cell senescence is involved in the pathological progression of DCI, but also found that ZSWF, a prescription for clearing heat, has the potential to improve DCI by preventing cerebrovascular cell senescence. Notably, recent accumulated studies have shown that cerebrovascular senescence can lead to the destruction of BBB integrity, and delaying cerebrovascular senescence or removing senescent cerebrovascular cells can restore BBB integrity [24, 25]. Meanwhile, recent studies have reported that diabetes can accelerate cerebral vascular senescent [27]. Accordingly, we conclude that ZSWF may restore BBB integrity in DCI mice by preventing cerebrovascular senescence. The detailed mechanism was reflected in that ZSWF reduced the expression of senescence markers *Cdkn1a* and SASP genes in cerebrovascular cells, prevented the senescence of cerebrovascular cells driven by diabetes environment, repaired BBB integrity, and thus improved the cognitive function of DCI mice.

Finally, to further discover the potential active components of ZSWF in anti-vascular senescence, we used the previously identified blood entry components as small molecules and the upstream protein of p21 as target proteins to predict them by molecular docking. Notably, mangiferin stands out from these small molecules, as reflected in better affinity between mangiferin and proteins (such as ERK, mTOR, P38, and MDM2). To confirm this finding, we cultured cerebral microvascular endothelial cells in vitro and established a high glucose injury model using 30 mM glucose. As expected, our results showed that high glucose significantly induced endothelial cell senescence, and mangiferin significantly inhibited the expression of senescence marker, confirming that mangiferin indeed has an anti-vascular senescence effect. Similar to the results reported in the literature, previous accumulated studies have also confirmed that mangiferin has excellent anti-senescence effects both in vivo and in vitro [72, 73]. These results suggest that mangiferin is a potential active component of ZSWF to repair BBB integrity in DCI mice by anti-vascular senescence. Overall, our study on the one hand reveals a new pathological mechanism of DCI in which the diabetic environment accelerates cerebrovascular senescence, leading to BBB integrity destruction and worsening cognitive impairment, on the other hand, it also elucidates the potential of ZSWF to repair BBB integrity and improve cognitive function in DCI mice by preventing cerebrovascular senescence. These findings not only provide a new strategy for the prevention and treatment of DCI, but also provide a theoretical basis for the clinical application of heat-clearing traditional chinese medicine. Notably, recent studies have proposed that panvascular injury is a common pathological event of diabetes-induced multi-organ complications [74, 75], and accumulated studies have also observed that diabetic environment accelerates vascular aging in multiple organs [27, 76, 77]. Therefore, we expect that panvascular senescence induced by diabetic environment may be the common pathological mechanism of diabetic multi-organ complications, and anti-vascular senescence may be a new strategy to delay or prevent the progression of diabetic complications.

That said, there are limitations to our current study. First, a suitable positive control was not selected for this study because there are no drugs with clear clinical efficacy against DCI. Second, the small molecules of ZSWF entering the blood used in the current study of molecular docking are prototype components, and the relationship between their metabolic components and senescence pathways has not been investigated. Third, although we preliminarily identified mangiferin as the active ingredient of ZSWF in anti-vascular senescence to repair BBB integrity in DCI mice, the detailed molecular pathways regulated by mangiferin have not yet been fully verified. Fourth, it is still unclear whether mangiferin can also repair BBB integrity and improve cognitive impairment in DCI mice by preventing cerebral vascular senescence in vivo. These limitations will be the focus of our future research.

## Conclusion

Overall, our study reveals that ZSWF can ameliorate cognitive function in DCI mice by repairing BBB integrity, and the specific mechanism of which may be related to preventing cerebrovascular cells senescence and improving abnormal communication between them, and mangiferin its key active ingredient. This study provides a new perspective for the development of DCI combination drugs for clinical prevention and treatment, and also provides theoretical support for the future clinical application of ZSWF.

## Data Availability

The original datasets used during the current study are available from the corresponding author on reasonable request.

## Abbreviations

AS: Astrocytes
BBB: Blood-brain-barrier
CPEC: Choroid plexus epithelial cells
DCI: Diabetes-induced cognitive impairment
ECs: Endothelial cells
EP: Ependymal cells
FB: Perivascular fibroblast-like cells
FBG: Fasting blood glucose
MG: Microglia
MWM: Morris water maze
Neur: Neurons
Neut: Neutrophils
OLG: Oligodendrocytes
OPC: Oligodendrocyte progenitor cells
PCs: Pericytes
Pdgfb: Platelet-derived growth factor B
scRNA-seq: Single-cell RNA sequencing
SASP: Senescence associated secretory phenotype
STZ: Streptozotocin
T/NK: T/Natural killer cells
VSMC: Vascular smooth muscle cells
ZSWF: Zi Shen Wan Fang

## References

[1] Baumgart M, Snyder HM, Carrillo MC, Fazio S, Kim H, Johns H. Summary of the evidence on modifiable risk factors for cognitive decline and dementia: a population-based perspective. Alzheimers Dement. 2015;11(6):718–26. 26045020 10.1016/j.jalz.2015.05.016

[2] Tuligenga RH, Dugravot A, Tabák AG, Elbaz A, Brunner EJ, Kivimäki M, et al. Midlife type 2 diabetes and poor glycaemic control as risk factors for cognitive decline in early old age: a post-hoc analysis of the Whitehall II cohort study. Lancet Diabetes Endocrinol. 2014;2(3):228–35. 24622753 10.1016/S2213-8587(13)70192-X PMC4274502

[3] Biessels GJ, Despa F. Cognitive decline and dementia in diabetes mellitus: mechanisms and clinical implications. Nat Rev Endocrinol. 2018;14(10):591–604. 30022099 10.1038/s41574-018-0048-7 PMC6397437

[4] Dove A, Shang Y, Xu W, Grande G, Laukka EJ, Fratiglioni L, et al. The impact of diabetes on cognitive impairment and its progression to dementia. Alzheimers Dement. 2021;17(11):1769–78. 34636485 10.1002/alz.12482

[5] Zilliox LA, Chadrasekaran K, Kwan JY, Russell JW. Diabetes and cognitive impairment. Curr Diab Rep. 2016;16(9):87. 27491830 10.1007/s11892-016-0775-x PMC5528145

[6] Weinstein G, Davis-Plourde KL, Conner S, Himali JJ, Beiser AS, Lee A, et al. Association of metformin, sulfonylurea and insulin use with brain structure and function and risk of dementia and Alzheimer’s disease: pooled analysis from 5 cohorts. PLoS ONE. 2019;14(2):e0212293. 30768625 10.1371/journal.pone.0212293 PMC6377188

[7] Craft S, Baker LD, Montine TJ, Minoshima S, Watson GS, Claxton A, et al. Intranasal insulin therapy for Alzheimer disease and amnestic mild cognitive impairment: a pilot clinical trial. Arch Neurol. 2012;69(1):29–38. 21911655 10.1001/archneurol.2011.233 PMC3260944

[8] Cukierman-Yaffe T, Bosch J, Diaz R, Dyal L, Hancu N, Hildebrandt P, et al. Effects of basal insulin glargine and omega-3 fatty acid on cognitive decline and probable cognitive impairment in people with dysglycaemia: a substudy of the ORIGIN trial. Lancet Diabetes Endocrinol. 2014;2(7):562–72. 24898834 10.1016/S2213-8587(14)70062-2

[9] Tuligenga RH. Intensive glycaemic control and cognitive decline in patients with type 2 diabetes: a meta-analysis. Endocr Connect. 2015;4(2):R16–24. 25712899 10.1530/EC-15-0004 PMC4419843

[10] Bahadar GA, Shah ZA. Intracerebral hemorrhage and diabetes mellitus: blood-brain barrier disruption, pathophysiology and cognitive impairments. CNS Neurol Disord: Drug Targets. 2021;20(4):312–26. 33622232 10.2174/1871527320666210223145112

[11] Brook E, Mamo J, Wong R, Al-Salami H, Falasca M, Lam V, et al. Blood-brain barrier disturbances in diabetes-associated dementia: therapeutic potential for cannabinoids. Pharmacol Res. 2019;141:291–7. 30616019 10.1016/j.phrs.2019.01.009

[12] Rom S, Zuluaga-Ramirez V, Gajghate S, Seliga A, Winfield M, Heldt NA, et al. Hyperglycemia-driven neuroinflammation compromises BBB leading to memory loss in both diabetes mellitus (DM) type 1 and type 2 mouse models. Mol Neurobiol. 2019;56(3):1883–96. 29974394 10.1007/s12035-018-1195-5 PMC6320739

[13] Lin L, Wu Y, Chen Z, Huang L, Wang L, Liu L. Severe hypoglycemia contributing to cognitive dysfunction in diabetic mice is associated with pericyte and blood-brain barrier dysfunction. Front Aging Neurosci. 2021;13:775244. 34899278 10.3389/fnagi.2021.775244 PMC8662820

[14] Wang S, Jiao F, Border JJ, Fang X, Crumpler RF, Liu Y, et al. Luseogliflozin, a sodium-glucose cotransporter-2 inhibitor, reverses cerebrovascular dysfunction and cognitive impairments in 18-mo-old diabetic animals. Am J Physiol Heart Circ Physiol. 2022;322(2):H246–59. 34951541 10.1152/ajpheart.00438.2021 PMC8759958

[15] Ward R, Li W, Abdul Y, Jackson L, Dong G, Jamil S, et al. NLRP3 inflammasome inhibition with MCC950 improves diabetes-mediated cognitive impairment and vasoneuronal remodeling after ischemia. Pharmacol Res. 2019;142:237–50. 30818045 10.1016/j.phrs.2019.01.035 PMC6486792

[16] Liebner S, Dijkhuizen RM, Reiss Y, Plate KH, Agalliu D, Constantin G. Functional morphology of the blood-brain barrier in health and disease. Acta Neuropathol. 2018;135(3):311–36. 29411111 10.1007/s00401-018-1815-1 PMC6781630

[17] Mäe MA, He L, Nordling S, Vazquez-Liebanas E, Nahar K, Jung B, et al. Single-cell analysis of blood-brain barrier response to pericyte loss. Circ Res. 2021;128(4):e46–62. 33375813 10.1161/CIRCRESAHA.120.317473 PMC10858745

[18] Jeong HW, Diéguez-Hurtado R, Arf H, Song J, Park H, Kruse K, et al. Single-cell transcriptomics reveals functionally specialized vascular endothelium in brain. eLife. 2022;11. 10.7554/eLife.57520 PMC9566870 36197007

[19] Xiao L, do Carmo LS, Foss JD, Chen W, Harrison DG. Sympathetic enhancement of memory T-cell homing and hypertension sensitization. Circul Res. 2020;126(6):708–21. 10.1161/CIRCRESAHA.119.314758 PMC8253247 31928179

[20] Wang J, Xu J, Zang G, Zhang T, Wu Q, Zhang H, et al. Trans-2-enoyl-CoA reductase tecr-driven lipid metabolism in endothelial cells protects against transcytosis to maintain blood-brain barrier homeostasis. Research. 2022;2022:9839368. 35465346 10.34133/2022/9839368 PMC9006154

[21] Ahire C, Nyul-Toth A, DelFavero J, Gulej R, Faakye JA, Tarantini S, et al. Accelerated cerebromicrovascular senescence contributes to cognitive decline in a mouse model of paclitaxel (Taxol)-induced chemobrain. Aging Cell. 2023;22(7):e13832. 37243381 10.1111/acel.13832 PMC10352561

[22] Vanlandewijck M, He L, Mäe MA, Andrae J, Ando K, Del Gaudio F, et al. A molecular atlas of cell types and zonation in the brain vasculature. Nature. 2018;554(7693):475–80. 29443965 10.1038/nature25739

[23] He S, Sharpless NE. Senescence in health and disease. Cell. 2017;169(6):1000–11. 28575665 10.1016/j.cell.2017.05.015 PMC5643029

[24] Yamazaki Y, Baker DJ, Tachibana M, Liu CC, van Deursen JM, Brott TG, et al. Vascular cell senescence contributes to blood-brain barrier breakdown. Stroke. 2016;47(4):1068–77. 26883501 10.1161/STROKEAHA.115.010835 PMC4811685

[25] Ya J, Kadir RRA, Bayraktutan U. Delay of endothelial cell senescence protects cerebral barrier against age-related dysfunction: role of senolytics and senomorphics. Tissue Barriers. 2023;11(3):2103353. 35880392 10.1080/21688370.2022.2103353 PMC10364655

[26] Salvador E, Burek M, Löhr M, Nagai M, Hagemann C, Förster CY. Senescence and associated blood-brain barrier alterations in vitro. Histochem Cell Biol. 2021;156(3):283–92. 34043058 10.1007/s00418-021-01992-z PMC8460501

[27] Phoenix A, Chandran R, Ergul A. Cerebral microvascular senescence and inflammation in diabetes. Front Physiol. 2022;13:864758. 35574460 10.3389/fphys.2022.864758 PMC9098835

[28] Bury JJ, Chambers A, Heath PR, Ince PG, Shaw PJ, Matthews FE, et al. Type 2 diabetes mellitus-associated transcriptome alterations in cortical neurones and associated neurovascular unit cells in the ageing brain. Acta Neuropathol Commun. 2021;9(1):5. 33407907 10.1186/s40478-020-01109-y PMC7788898

[29] Huang J, Lin W, Sun Y, Wang Q, He S, Han Z, et al. Quercetin targets VCAM1 to prevent diabetic cerebrovascular endothelial cell injury. Front Aging Neurosci. 2022;14:944195. 36118693 10.3389/fnagi.2022.944195 PMC9475220

[30] Shi J, Yin Q, Zhang L, Wu Y, Yi P, Guo M, et al. Zi Shen Wan Fang attenuates neuroinflammation and cognitive function via remodeling the gut microbiota in diabetes-induced cognitive impairment mice. Front Pharmacol. 2022;13:898360. 35910371 10.3389/fphar.2022.898360 PMC9335489

[31] Yin Q, Zhang L, Han X, Zhang H, Wang F, Qin X, et al. Zi Shen Wan Fang regulates kynurenine metabolism to alleviate diabetes-associated cognitive impairment via activating the skeletal muscle PGC1α-PPARα signaling. Phytomedicine. 2022;99:154000. 35235888 10.1016/j.phymed.2022.154000

[32] Zheng Y, Zhang Y, Geng S, Xu M, Yin Q, Song L, et al. Identification of the constituents and metabolites in rats after oral administration of Zi Shen Formula by UPLC-Q-TOF/MS combined pattern recognition analysis. Biomed Chromatogr. 2018;32(2). 10.1002/bmc.4060 28793175

[33] Li X, Yin Q, Han X, Zhang H, Wang F, Ma J, et al. Dynamic expression of vascular endothelial growth factor (VEGF) and platelet-derived growth factor receptor beta (PDGFRβ) in diabetic brain contributes to cognitive dysfunction. Brain Res Bull. 2021;175:99–106. 34303767 10.1016/j.brainresbull.2021.07.017

[34] Morris R. Developments of a water-maze procedure for studying spatial learning in the rat. J Neurosci Methods. 1984;11(1):47–60. 6471907 10.1016/0165-0270(84)90007-4

[35] Geng J, Wang L, Zhang L, Qin C, Song Y, Ma Y, et al. Blood-brain barrier disruption induced cognitive impairment is associated with increase of inflammatory cytokine. Front Aging Neurosci. 2018;10:129. 29867440 10.3389/fnagi.2018.00129 PMC5949351

[36] Zhang R, Lahens NF, Ballance HI, Hughes ME, Hogenesch JB. A circadian gene expression atlas in mammals: implications for biology and medicine. Proc Natl Acad Sci USA. 2014;111(45):16219–24. 25349387 10.1073/pnas.1408886111 PMC4234565

[37] Butler A, Hoffman P, Smibert P, Papalexi E, Satija R. Integrating single-cell transcriptomic data across different conditions, technologies, and species. Nat Biotechnol. 2018;36(5):411–20. 29608179 10.1038/nbt.4096 PMC6700744

[38] McGinnis CS, Murrow LM, Gartner ZJ. DoubletFinder: doublet detection in single-cell RNA sequencing data using artificial nearest neighbors. Cell Syst. 2019;8(4):329-37.e4. 30954475 10.1016/j.cels.2019.03.003 PMC6853612

[39] Yang S, Corbett SE, Koga Y, Wang Z, Johnson WE, Yajima M, et al. Decontamination of ambient RNA in single-cell RNA-seq with DecontX. Genome Biol. 2020;21(1):57. 32138770 10.1186/s13059-020-1950-6 PMC7059395

[40] Liu B, Li C, Li Z, Wang D, Ren X, Zhang Z. An entropy-based metric for assessing the purity of single cell populations. Nat Commun. 2020;11(1):3155. 32572028 10.1038/s41467-020-16904-3 PMC7308400

[41] Efremova M, Vento-Tormo M, Teichmann SA, Vento-Tormo R. Cell PhoneDB: inferring cell-cell communication from combined expression of multi-subunit ligand-receptor complexes. Nat Protoc. 2020;15(4):1484–506. 32103204 10.1038/s41596-020-0292-x

[42] Ximerakis M, Lipnick SL, Innes BT, Simmons SK, Adiconis X, Dionne D, et al. Single-cell transcriptomic profiling of the aging mouse brain. Nat Neurosci. 2019;22(10):1696–708. 31551601 10.1038/s41593-019-0491-3

[43] Allen WE, Blosser TR, Sullivan ZA, Dulac C, Zhuang X. Molecular and spatial signatures of mouse brain aging at single-cell resolution. Cell. 2023;186(1):194-208.e18. 36580914 10.1016/j.cell.2022.12.010 PMC10024607

[44] Zhong J, Tang G, Zhu J, Wu W, Li G, Lin X, et al. Single-cell brain atlas of Parkinson’s disease mouse model. J Genet Genomics. 2021;48(4):277–88. 34052184 10.1016/j.jgg.2021.01.003

[45] Prakash R, Somanath PR, El-Remessy AB, Kelly-Cobbs A, Stern JE, Dore-Duffy P, et al. Enhanced cerebral but not peripheral angiogenesis in the Goto-Kakizaki model of type 2 diabetes involves VEGF and peroxynitrite signaling. Diabetes. 2012;61(6):1533–42. 22403298 10.2337/db11-1528 PMC3357273

[46] Abdelsaid M, Coucha M, Hafez S, Yasir A, Johnson MH, Ergul A. Enhanced VEGF signalling mediates cerebral neovascularisation via downregulation of guidance protein ROBO4 in a rat model of diabetes. Diabetologia. 2017;60(4):740–50. 28116460 10.1007/s00125-017-4214-6 PMC5342922

[47] Zlokovic BV. Neurovascular pathways to neurodegeneration in Alzheimer’s disease and other disorders. Nat Rev Neurosci. 2011;12(12):723–38. 22048062 10.1038/nrn3114 PMC4036520

[48] Rom S, Heldt NA, Gajghate S, Seliga A, Reichenbach NL, Persidsky Y. Hyperglycemia and advanced glycation end products disrupt BBB and promote occludin and claudin-5 protein secretion on extracellular microvesicles. Sci Rep. 2020;10(1):7274. 32350344 10.1038/s41598-020-64349-x PMC7190636

[49] Foidl BM, Humpel C. Chronic treatment with five vascular risk factors causes cerebral amyloid angiopathy but no Alzheimer pathology in C57BL6 mice. Brain Behav Immun. 2019;78:52–64. 30664922 10.1016/j.bbi.2019.01.009

[50] Hawkins BT, Lundeen TF, Norwood KM, Brooks HL, Egleton RD. Increased blood-brain barrier permeability and altered tight junctions in experimental diabetes in the rat: contribution of hyperglycaemia and matrix metalloproteinases. Diabetologia. 2007;50(1):202–11. 17143608 10.1007/s00125-006-0485-z

[51] Kumari R, Bettermann K, Willing L, Sinha K, Simpson IA. The role of neutrophils in mediating stroke injury in the diabetic db/db mouse brain following hypoxia-ischemia. Neurochem Int. 2020;139:104790. 32652270 10.1016/j.neuint.2020.104790

[52] Habib N, Avraham-Davidi I, Basu A, Burks T, Shekhar K, Hofree M, et al. Massively parallel single-nucleus RNA-seq with DroNc-seq. Nat Methods. 2017;14(10):955–8. 28846088 10.1038/nmeth.4407 PMC5623139

[53] Lake BB, Ai R, Kaeser GE, Salathia NS, Yung YC, Liu R, et al. Neuronal subtypes and diversity revealed by single-nucleus RNA sequencing of the human brain. Science. 2016;352(6293):1586–90. 27339989 10.1126/science.aaf1204 PMC5038589

[54] Zhong S, Zhang S, Fan X, Wu Q, Yan L, Dong J, et al. A single-cell RNA-seq survey of the developmental landscape of the human prefrontal cortex. Nature. 2018;555(7697):524–8. 29539641 10.1038/nature25980

[55] Mathys H, Davila-Velderrain J, Peng Z, Gao F, Mohammadi S, Young JZ, et al. Single-cell transcriptomic analysis of Alzheimer’s disease. Nature. 2019;570(7761):332–7. 31042697 10.1038/s41586-019-1195-2 PMC6865822

[56] Blanchard JW, Akay LA, Davila-Velderrain J, von Maydell D, Mathys H, Davidson SM, et al. APOE4 impairs myelination via cholesterol dysregulation in oligodendrocytes. Nature. 2022;611(7937):769–79. 36385529 10.1038/s41586-022-05439-w PMC9870060

[57] Liu P, Qi G, Gu S, Dong H, Liu C, Yang H. Single-cell transcriptomics and network pharmacology reveal therapeutic targets of Jianpi Yiqi Bugan Yishen decoction in immune cell subsets of children with myasthenia gravis. Transl Pediatr. 2022;11(12):1985–2003. 36643680 10.21037/tp-22-593 PMC9834954

[58] Liu S, Cao X, Zhang T, Zhang C, Qu J, Sun Y, et al. Paeonol ameliorates endometrial hyperplasia in mice via inhibiting PI3K/AKT pathway-related ferroptosis. Phytomedicine. 2023;109:154593. 36610113 10.1016/j.phymed.2022.154593

[59] Ren W, Ban J, Xia Y, Zhou F, Yuan C, Jia H, et al. Echinacea purpurea-derived homogeneous polysaccharide exerts anti-tumor efficacy via facilitating M1 macrophage polarization. Innovation. 2023;4(2):100391. 36873268 10.1016/j.xinn.2023.100391 PMC9974447

[60] Yu D, Yang P, Lu X, Huang S, Liu L, Fan X. Single-cell RNA sequencing reveals enhanced antitumor immunity after combined application of PD-1 inhibitor and Shenmai injection in non-small cell lung cancer. Cell Commun Signal. 2023;21(1):169. 37430270 10.1186/s12964-023-01184-3 PMC10332015

[61] Zeisel A, Muñoz-Manchado AB, Codeluppi S, Lönnerberg P, La Manno G, Juréus A, et al. Brain structure. Cell types in the mouse cortex and hippocampus revealed by single-cell RNA-seq. Science. 2015;347(6226):1138–42. 10.1126/science.aaa1934 25700174

[62] Kiss T, Nyúl-Tóth Á, Balasubramanian P, Tarantini S, Ahire C, DelFavero J, et al. Single-cell RNA sequencing identifies senescent cerebromicrovascular endothelial cells in the aged mouse brain. GeroScience. 2020;42(2):429–44. 32236824 10.1007/s11357-020-00177-1 PMC7205992

[63] Zhang P, Kishimoto Y, Grammatikakis I, Gottimukkala K, Cutler RG, Zhang S, et al. Senolytic therapy alleviates Aβ-associated oligodendrocyte progenitor cell senescence and cognitive deficits in an Alzheimer’s disease model. Nat Neurosci. 2019;22(5):719–28. 30936558 10.1038/s41593-019-0372-9 PMC6605052

[64] Choi I, Wang M, Yoo S, Xu P, Seegobin SP, Li X, et al. Autophagy enables microglia to engage amyloid plaques and prevents microglial senescence. Nat Cell Biol. 2023;25(7):963–74. 37231161 10.1038/s41556-023-01158-0 PMC10950302

[65] Long C, Liu H, Zhan W, Chen L, Yu Z, Tian S, et al. Chronological attenuation of NPRA/PKG/AMPK signaling promotes vascular aging and elevates blood pressure. Aging Cell. 2022;21(9):e13699. 36016499 10.1111/acel.13699 PMC9470896

[66] Xu P, Wang M, Song WM, Wang Q, Yuan GC, Sudmant PH, et al. The landscape of human tissue and cell type specific expression and co-regulation of senescence genes. Mol Neurodegener. 2022;17(1):5. 35000600 10.1186/s13024-021-00507-7 PMC8744330

[67] Xiang J, Shen J, Zhang L, Tang B. Identification and validation of senescence-related genes in circulating endothelial cells of patients with acute myocardial infarction. Front Cardiovasc Med. 2022;9:1057985. 36582740 10.3389/fcvm.2022.1057985 PMC9792765

[68] Liu S, Zhang Z, He Y, Kong L, Jin Q, Qi X, et al. Inhibiting leukocyte-endothelial cell interactions by Chinese medicine Tongxinluo capsule alleviates no-reflow after arterial recanalization in ischemic stroke. CNS Neurosci Ther. 2023;29(10):3014–30. 37122157 10.1111/cns.14242 PMC10493667

[69] Voirin AC, Perek N, Roche F. Inflammatory stress induced by a combination of cytokines (IL-6, IL-17, TNF-α) leads to a loss of integrity on bEnd.3 endothelial cells in vitro BBB model. Brain Res. 2020;1730:146647. 10.1016/j.brainres.2020.146647 31911168

[70] Bhowmick S, D’Mello V, Caruso D, Wallerstein A, Abdul-Muneer PM. Impairment of pericyte-endothelium crosstalk leads to blood-brain barrier dysfunction following traumatic brain injury. Exp Neurol. 2019;317:260–70. 30926390 10.1016/j.expneurol.2019.03.014

[71] Shen J, Xu G, Zhu R, Yuan J, Ishii Y, Hamashima T, et al. PDGFR-β restores blood-brain barrier functions in a mouse model of focal cerebral ischemia. J Cereb Blood Flow Metab. 2019;39(8):1501–15. 29629621 10.1177/0271678X18769515 PMC6681529

[72] Chomchoei N, Leelapornpisid P, Tipduangta P, Sangthong P, Papan P, Sirithunyalug B, et al. Potential of electro-sprayed purified mangiferin nanoparticles for anti-aging cosmetic applications. RSC Adv. 2023;13(50):34987–5002. 38046636 10.1039/d3ra06308a PMC10690135

[73] Kanoi R, Loachan P, Das S, Rao BSS. Mangiferin, a naturally occurring polyphenol, mitigates oxidative stress induced premature senescence in human dermal fibroblast cells. Mol Biol Rep. 2021;48(1):457–66. 33393007 10.1007/s11033-020-06074-2

[74] Li Y, Liu Y, Liu S, Gao M, Wang W, Chen K, Huang L, Liu Y. Diabetic vascular diseases: molecular mechanisms and therapeutic strategies. Signal Transduct Target Ther. 2023;8(1):152. 37037849 10.1038/s41392-023-01400-z PMC10086073

[75] Madonna R, Balistreri CR, Geng YJ, De Caterina R. Diabetic microangiopathy: pathogenetic insights and novel therapeutic approaches. Vascul Pharmacol. 2017;90:1–7. 28137665 10.1016/j.vph.2017.01.004

[76] Liu H, Ghosh S, Vaidya T, Bammidi S, Huang C, Shang P, et al. Activated cGAS/STING signaling elicits endothelial cell senescence in early diabetic retinopathy. Am Soc Clin Investig. 2023;8(12):e168945. 10.1172/jci.insight.168945 PMC10371250 37345657

[77] Nian S, Mi Y, Ren K, Wang S, Li M, Yang D. The inhibitory effects of Dulaglutide on cellular senescence against high glucose in human retinal endothelial cells. Hum Cell. 2022;35(4):995–1004. 35583801 10.1007/s13577-022-00703-7

